# Rotenone and Its Derivative, Rotenoisin A, Induce Neurodegeneration Differentially in SH-SY5Y Cells

**DOI:** 10.3390/biomedicines12081703

**Published:** 2024-07-31

**Authors:** Mahesh Ramalingam, Sujeong Jang, Seongryul Kim, Hyoungwoo Bai, Gyeonghan Jeong, Byeong C. Kim, Han-Seong Jeong

**Affiliations:** 1 Department of Physiology, Chonnam National University Medical School, Hwasun 58128, Republic of Korea; sujeong.jjang@gmail.com (S.J.); ryul5204@hanmail.net (S.K.); 2 Department of Radiation Science, Korea Atomic Energy Research Institute (KAERI), Jeongeup 56212, Republic of Korea; hbai@kaeri.re.kr (H.B.); jkh4598@kaeri.re.kr (G.J.); 3 Radiation Biotechnology and Applied Radioisotope Science, University of Science and Technology (UST), Daejeon 34113, Republic of Korea; 4 Department of Neurology, Chonnam National University Medical School, Gwangju 61469, Republic of Korea; byeong.kim7@gmail.com; 5 Department of Neurology, Chonnam National University Hospital, Gwangju 61469, Republic of Korea

**Keywords:** α-synuclein, neurotoxicity, autophagy, Akt, caspases

## Abstract

Rotenone (ROT), the most significant rotenoid, which has shown anticancer activity, has also been reported to be toxic to normal cells, inducing Parkinson’s disease (PD)-like neuronal loss with aggregation of α-synuclein (α-syn). To reduce the adverse effects of ROT, its derivative, rotenoisin A (ROA), is obtained by directly irradiating a ROT solution in methanol using γ-rays, which has been reported for potential anticancer properties. However, its PD-inducing effects have not yet been researched or reported. This study sought to compare the activities of ROA and ROT on the aggregation of α-syn, apoptosis, and autophagy in SH-SY5Y cells. ROA decreased cell survival less when compared with ROT on SH-SY5Y cells at 48 h in a dose-dependent manner. ROT (0.5 and 1 μM) and ROA (4 and 5 μM) decreased the expression of tyrosine hydroxylase. Western blot analysis of the Triton X-100 insoluble fraction revealed that both ROT and ROA significantly increased the levels of oligomeric, dimeric, and monomeric phosphorylated Serine129 α-syn and total monomeric α-syn. Moreover, both compounds decreased the proportion of neuronal nuclei, the neurofilament-heavy chain, and β3-tubulin. The phosphorylation of ERK and SAPK were reduced, whereas ROA did not act on Akt. Additionally, the increased Bax/Bcl-2 ratio further activated the downstream caspases cascade. ROT promoted the LC3BII/I ratio and p62 levels; however, different ROA doses resulted in different effects on autophagy while inducing PD-like impairments in SH-SY5Y cells.

## 1. Introduction

Rotenoids are natural compounds found in various leguminous plants including the genera *Derris*, *Lonchocarpus*, and *Tephrosia*, among others, and have been utilized as fish poison for many centuries [1]. Rotenoids are commonly extracted using methanol or ethanol as a solvent system to achieve more accurate efficiency and higher yields [2]. These compounds belong to the isoflavonoid family, containing an extra carbon at the C-2 position connected to the C-2’ position through an ether linkage to form a tetracyclic ring system [3]. Rotenoids inhibit the mitochondrial oxidation of nicotinamide adenine dinucleotide (NADH) and serve as insecticides with known cytotoxic properties against several cancer cell lines [4,5,6].

Rotenone (ROT) is an important rotenoid derived from *Derris elliptica* and *D. malaccensis* in Asia, as well as *Lonchocarpus utilis* and *L. urucu* in South America [1]. It is primarily used as a piscicide (poisonous to fish) worldwide, being almost insoluble in water but readily soluble in many organic solvents [7]. ROT undergoes hydroxylation at C-7 when exposed to light and air, followed by dehydration and decomposition by alkaline pH [7]. Biochemically, ROT is a potent inhibitor of the NADH dehydrogenase system, acting on iron–sulfur (Fe-S) proteins to constrain electron transport between NADH and ubiquinone (coenzyme Q) [1]. Electron translocation leads to the degradation of the proton-motive force, reducing the ability to produce adenosine triphosphate (ATP), resulting in mitochondrial dysfunction. This contributes to the release of proapoptotic factors, leading to cellular dysfunction and death [8].

ROT contains a central dihydro-γ-pyrone ring with dihydrobenzopyran and dihydrobenzofuran systems. However, the central hexagonal rings contribute to mitochondrial toxicity [9], which is suggested to have an anticancer mode of action [10] against several cancer cell lines. ROT reduced the tongue squamous cell carcinoma during the post-initiation phase of 4-nitroquinoline 1-oxide (4-NQO)-induced tongue tumorigenesis in male F344 rats [11]. ROT induced higher toxicity in oral cancer cells than in normal oral mucosal fibroblasts [12]. Furthermore, ROT increased reactive oxygen species (ROS) and MAPKs activation, which leads to inducing apoptosis in human breast cancer MCF-7 cells [13]. Low doses of ROT stimulated mammosphore formation; however, higher doses inhibited mammosphore formation due to toxic levels of oxidative stress in MCF-7 cells, showing unnecessary adverse effects [14].

ROT has been reported to be toxic to normal cells [6], which has been linked to neurological disorders and the onset of Parkinson’s disease (PD)-like impairments. A study reported that the agricultural use of ROT is associated with a 2.5 times higher incidence of PD compared to non-users in humans [15]. Previous studies have revealed that ROT causes mitochondrial dysfunction, decreases the activity of tyrosine hydroxylase, increases the phosphorylation and aggregation of α-synuclein (α-syn), impairs autophagy degradation, and triggers endoplasmic reticulum (ER) stress, ultimately upregulating apoptotic pathways and inducing cellular death in SH-SY5Y neuroblastoma cells [16,17,18]. These findings underscore the need to explore the structure–activity relationship of rotenoids to mitigate the risk of neurotoxicity to mammals and reduce detrimental effects on the environment [19]. Nevertheless, very few studies have explored the changes in the chemical structure of ROT to produce derivatives [6,20], nor have there been substantial efforts to characterize the toxic effects of these derivatives.

**Figure 1:**
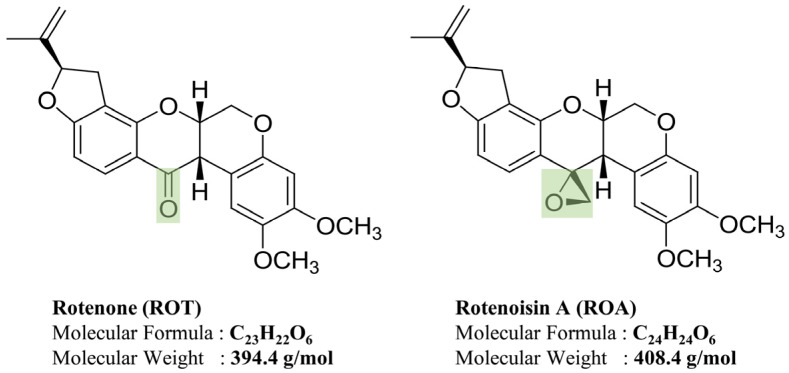
Chemical structures of ROT and ROA.

Studies have previously reported the generation of ROT derivates from ROT through radiolytic transformation. ROT in methanol, irradiated directly using γ-rays, leads to the derivative rotenoisin A (ROA), with a replacement of the ketone moiety at the C-12 position by an epoxy ring [21] (Figure 1 and Supplementary Figure S1). ROA has shown enhanced anti-adipogenic effects by potently inhibiting pancreatic lipase activity and 3T3-L1 adipocytes compared to ROT [21]. Moreover, various concentrations of ROA during an 8-day differentiation period significantly inhibited 3T3-L1 adipogenesis and targeted associated pathways, presenting a therapeutic potential for obesity and related disorders [22]. ROA treatment in primary epidermal keratinocytes inhibited breast cancer cell proliferation and enhanced apoptosis [23]. Human neuroblastoma SH-SY5Y cells have been widely used as a model for investigating neurodegenerative disorders, such as PD [16,17,18]. Therefore, this study sought to elucidate the insights into the several mechanisms through cytotoxic and inhibitory activities of ROA on SH-SY5Y cells by comparing them with the effects of its parent compound ROT while inducing PD-like impairments.

## 2. Materials and Methods

### 2.1. Cell Culture

The SH-SY5Y neuroblastoma cell line used in the study was obtained from the American Type Culture Collection (ATCC^®^ CRL-2266; Manassas, VA, USA). The cells were cultured in Dulbecco’s modified Eagle’s medium (DMEM; Welgene Inc., Gyeongsan, Republic of Korea) supplemented with 1% penicillin/streptomycin (Gibco BRL, Grand Island, NY, USA) and 10% (*v*/*v*) fetal bovine serum (FBS; Hyclone, Logan, UT, USA). The cells were maintained at 37 °C in a humidified incubator with 5% CO_2_. The culture medium was refreshed every 48 to 72 h, and passages 15–22 with a growth density of 75–85% were utilized for the experiments. To initiate the experimental studies, cultured cells were washed with phosphate-buffered saline (PBS), detached using 0.25% trypsin–EDTA solution, and then reseeded at a density of 50,000 cells/mL in DMEM with 1% FBS. The cells were then incubated overnight (~16 h). Then, 95% of the medium was replaced with fresh DMEM + 1% FBS medium before conducting further experiments.

### 2.2. ROT and ROA Preparation

ROA, a derivative of ROT, was synthesized at the Korea Atomic Energy Research Institute (KAERI, Jeongeup, Republic of Korea), as reported previously [21,23]. Briefly, ROT (0.5 g) in methanol (200 mL) in capped vials was exposed to 50 kGy of radiation at ambient temperature using a cobalt-60 irradiator. The irradiated solution was immediately evaporated to remove the solvent, lyophilized, and subjected to column chromatography over a silica gel column with CHCl_3_-MeOH-MeOH to yield pure ROA (129.1 mg).

ROT (R8875, MW 394.42, Sigma Chemical Co., St. Louis, MO, USA) or ROA (MW 408.4, KAERI) stock solutions at a concentration of 10 mM were prepared in a polar aprotic solvent, dimethyl sulfoxide (DMSO; D2650, Sigma). These stock solutions were aliquoted and stored at −80 °C, and they were used for experiments within six months. Working solutions of toxins at a concentration of 250 μM were prepared using DMEM without FBS before each experiment. Any remaining working solutions were discarded.

### 2.3. Cell Viability Assay

SH-SY5Y cells were treated in the presence of ROT or ROA at different concentrations for 48 h. Control cells were treated with DMSO. Phase contrast images were taken using an inverted microscope (Axio Vert.A1; Carl Zeiss Microscopy GmbH, Jena, Germany) equipped with a camera connected to i-Solution FL/Auto 10.0 software (IMT i-Solution Inc., Vancouver, BC, Canada) at the end of the experiment to analyze cell morphology. Damaged and detached floating cells in the medium and trypsinized cells were combined and the cell suspension was diluted to a 1:1 ratio with 0.4% (*v*/*v*) trypan blue staining solution (T8154, Sigma) for cell viability assays. The surviving cell numbers were counted using a LUNA-II^TM^ (Logos Biosystems, Anyang, Republic of Korea) mechanized cell counter. A cell count assay was performed in triplicate and expressed as a percentage (%) of the control.

### 2.4. Triton X-100 Soluble and Insoluble Cell Lysate Fractions

Western blotting was performed as described previously [16,17,18]. Briefly, cells were collected by scraping with culture medium and washed with PBS by centrifugation. This procedure was repeated twice. Then, the cells were lysed with Triton X-100 soluble cell lysis buffer containing rapid immunoprecipitation assay (RIPA) buffer (#89901, Thermo Scientific, Rockford, IL, USA), Halt protease inhibitor cocktail (#87789, Thermo Scientific), Halt phosphatase inhibitor cocktail (#78420, Thermo Scientific), and 1% Triton X-100 (X100, Sigma). The lysed cells, incubated for 30 min on ice at 8 °C, were centrifuged at 13,200 rpm (16,000× *g*) for 20 min at 4 °C, and the cell lysate supernatants were collected as Triton X-100 soluble fractions or whole cell lysates. The remaining pellets were washed with PBS, dissolved in a Triton X-100 insoluble cell lysis buffer (RIPA + protease and phosphate inhibitors + 1% Triton X-100 + 2% sodium dodecyl sulfate (SDS, L3771, Sigma), and sonicated for three minutes on ice. A pulsed duty cycle of 2 s on/2 s off was used with a power supply of 25%. The resulting suspensions were used as Triton X-100 insoluble fractions (2% SDS-soluble fractions).

### 2.5. Western Blotting

The protein levels were estimated using the BCA Protein Assay Kit (#23225, Thermo Scientific), and the same amounts (10 μg) of triton X-100-soluble cell lysates were loaded on 7–12% SDS–polyacrylamide gels. The proteins were separated according to their molecular weight in the gels and were transferred onto polyvinylidene difluoride (PVDF) membranes (Immobilon-P; IPVH00010, Merck Millipore, Bradford, MA, USA). The membranes were then immediately blocked with 5% nonfat dried milk or 1% bovine serum albumin (BSA) dissolved in 0.1% Tween 20 containing tris-buffered saline (TBS-T) washing buffer (pH 7.6). The membranes were then serially incubated with primary and secondary antibodies. The antibodies used (acquired from Merck Millipore, Temecula, CA, USA; Abcam, Cambridge, MA, USA; Cell Signaling Technology Inc., Danvers, MA, USA; and Santa Cruz Biotechnology, Santa Cruz, CA, USA) are listed in Supplementary Table S1. Lastly, the bands were imaged using an enhanced chemiluminescence (ECL) system (WBLUR0500, Millipore, Billerica, MA, USA) and a luminescent image analyzer (LAS 4000, GE Healthcare, Little Chalfont, UK). After imaging the phosphorylated proteins, the membranes were stripped with Western Blot Stripping Buffer (#21059, Thermo Scientific) and subsequently used to detect their corresponding total protein forms. GAPDH or β-actin were used to standardize the target protein levels. Phospho-protein signals were standardized against the corresponding total (non-phosphorylated) forms of the same target protein.

### 2.6. Evaluating the Expression of α-Syn

Triton X-100 soluble and insoluble (2% SDS soluble) lysate fractions were used to perform Western blotting to assess the α-syn aggregation by toxicity of ROT or ROA. The same amounts (20 μg) of the proteins were separated on 11–12% SDS-polyacrylamide gels and transferred onto nitrocellulose membranes (Immobilon-NC; HATF00010; Merck Millipore). After transfer, the membranes were immediately pre-fixed at room temperature for 1 h with 4% paraformaldehyde (PFA) in PBS containing 0.01% glutaraldehyde (340855, Sigma) to enhance the antibody binding sensitivity. Then, PBS-washed membranes were blocked with 5% skim milk in TBS-T for 1 h. The membranes were then serially incubated with anti-p-S129 α-syn (1:1000, ab51253, Abcam) primary antibodies overnight at 4 °C, followed by secondary antibodies at room temperature for 3 h. TBS-washed membranes were visualized for signals using ECL. After detecting the p-S129 α-syn, the membranes were washed and stripped with Western blot Stripping Buffer for 1 h. After being washed with PBS-T, the membranes were pre-fixed with 4% PFA in PBS for 1 h at room temperature and then rinsed with PBS. Blocking was performed with 5% skim milk in TBS-T for 1 h, and then this was incubated with total α-syn (1:1000, ab212184, Abcam) primary antibody overnight at 4 °C. Subsequently, the washed membranes were incubated with secondary antibodies diluted in blocking buffer. Next, the membranes were washed in TBS three times, each for 10 min, and the signals were imagined using ECL. GAPDH was used to standardize the levels of protein expressions.

### 2.7. Immunofluorescence (IF)

The IF protocol for suspension cells was used due to cell detachment during ROT or ROA toxicity. Briefly, the cells were collected by scraping with culture medium in a 15 mL conical tube followed by a centrifugation for 5 min. Afterwards, the cells were washed twice with 1× PBS by centrifugation to remove cell culture medium. Cells were immediately fixed at room temperature for 15 min with 4% paraformaldehyde in PBS at pH 7.4. After three washings in PBS, the cells were permeabilized with 0.1% Triton X-100 solution in PBS for 15 min. Next, the cells were blocked for 60 min at room temperature with 1% BSA in 0.1% Triton X-100 solution, followed by incubation with TH primary antibody (1:1000, AB152, Merck Millipore) diluted in blocking buffer for overnight at 4 °C. The cells were then washed three times with PBS and the binding of secondary antibody conjugated with Alexa Fluor 594 (1:500, A-11012, Invitrogen, Eugene, OR, USA) in blocking buffer kept in the dark for 2 h at room temperature. For staining nuclei, 4′,6-diamidino-2-phenylindole (DAPI, D-1306, Invitrogen) was added at the concentration of 1 μg/mL and incubated for 15 min. Further washing with PBS, the cells were mixed with Vectashield mounting medium for Fluorescence (H-1000, Vector Laboratories, Carpinteria, CA, USA) dropped on the microscopic slides and coverslips. Images were acquired at 40× using an ImageXpress Nano (Molecular Devices, San Jose, CA, USA). Raw images were exported with MetaXpress v6 (Molecular Devices), and overlay images were merged.

### 2.8. Statistical Analysis

All data were presented as the mean ± standard error of the mean (SEM) of three independent cell culture experiments. The ImageJ 1.54f densitometric analysis software (National Institutes of Health, Bethesda, MD, USA) was used to extract data from the images. Microsoft 365 Excel (Microsoft Corporation, Redmond, WA, USA) was used for data processing and GraphPad Prism^®^ 5.0 software (GraphPad Software Inc., San Diego, CA, USA) was used for statistical comparisons and for the generation of bar charts. One-way ANOVA followed by Tukey’s post hoc test or two-way ANOVA followed by Bonferroni post hoc test were performed to identify differences between experimental groups. *p*-values < 0.05 were considered statistically significant.

## 3. Results

### 3.1. Effects of ROT- or ROA-Induced Cell Death in SH-SY5Y Cells

**Figure 2:**
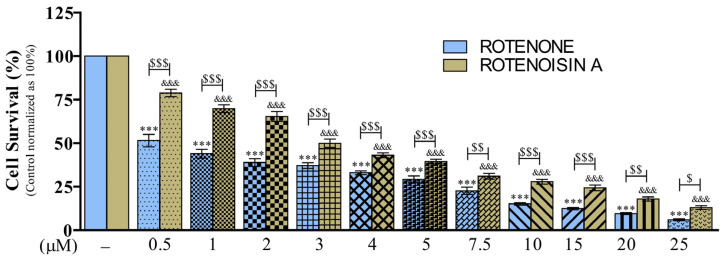
ROT- or ROA-induced cell death. SH-SY5Y cells were seeded at a density of 50,000 cells/mL in DMEM containing 1% FBS and used for experiments after overnight incubation. Cells were incubated with different concentrations of ROT and ROA and the percentage of live cells after 48 h of toxicity was evaluated by trypan blue assay. The data are expressed as mean ± SEM with n = 3 and analyzed by one-way analysis of variance (ANOVA) followed by Tukey’s post hoc test. *** *p* < 0.001 vs. control for ROT [F_(11,60)_ = 206.8, *p* < 0.0001, R^2^ = 0.9743]; ^&&&^ *p* < 0.001 vs. control for ROA [F_(11,60)_ = 233.5, *p* < 0.0001, R^2^ = 0.9772]. A two-way ANOVA followed by a Bonferroni post hoc test analyzed for the comparisons between ROT and ROA [row factor F_(11,120)_ = 428.1, *p* < 0.0001; column factor F_(1,120)_ = 347.9, *p* < 0.0001; interaction F_(11,120)_ = 11.93, *p* <0.0001]. ^$^ *p* < 0.05, ^$$^ *p* < 0.01, and ^$$$^ *p* < 0.001.

To assess the toxicity of ROT and ROA on cell viability, SH-SY5Y cells were cultured in DMEM with 1% FBS medium and incubated with different concentrations (0, 0.5, 1, 2, 3, 4, 5, 7.5, 10, 15, 20, and 25 μM) of ROT or ROA for 48 h (Figure 2 and Supplementary Figure S2). The Trypan blue cell counting assay, combining both adherent and floating cells, revealed that the cell viability percentage gradually decreased with ROT or ROA in a concentration-dependent manner upon exposure to all of the assayed concentrations compared with the control. According to the results of two-way analysis of variance (ANOVA), ROT significantly decreased the cell survival compared to those of the aforementioned ROA concentrations (Figure 2). ROT at concentrations of 0.5 and 1.0 μM reduced cell viability to 51.47 ± 3.57% and 43.99 ± 2.54%, respectively. However, ROA toxicity at 3.0 μM (49.98 ± 2.38), 4.0 μM (43.06 ± 1.36), and 5.0 μM (39.44 ± 1.39) reduced cell survival to levels comparable to ROT. Based on the outcomes of this experiment, all downstream experiments were conducted with ROA at 1, 2, 3, 4, and 5 μM compared to ROT at 0.5 and 1 μM (Supplementary Figure S3A). Morphological changes were also observed (Supplementary Figure S3B).

### 3.2. Effects of ROT or ROA on TH and DJ-1 Protein Expressions in SH-SY5Y Cells

**Figure 3:**
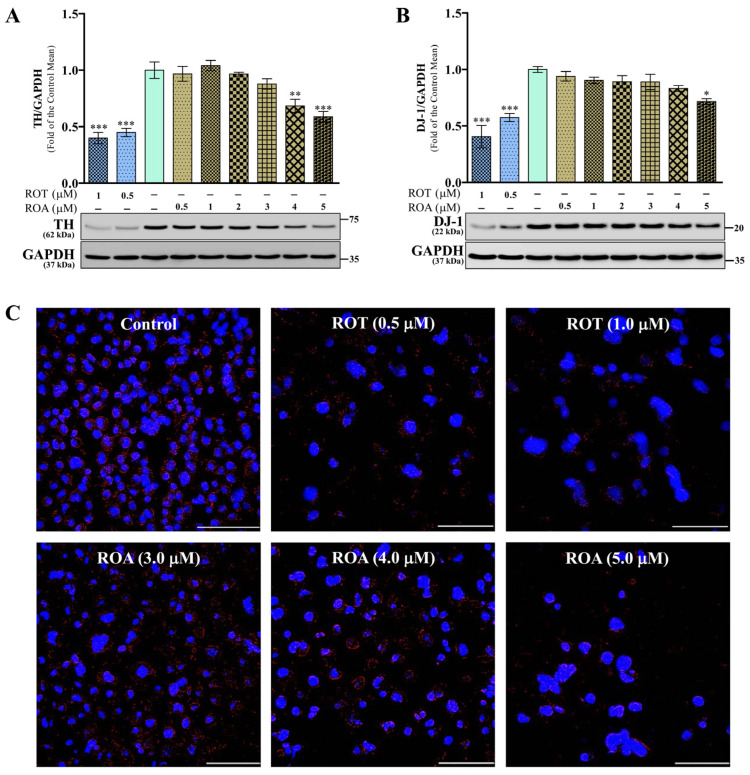
ROT or ROA on TH and DJ-1. SH-SY5Y cells were seeded at a density of 50,000 cells/mL in DMEM containing 1% FBS and used for experiments after overnight incubation. (**A**,**B**) Cells incubated with ROT or ROA for 48 h were collected, cell lysates were prepared, and the levels of TH/glyceraldehyde 3-phosphate dehydrogenase (GAPDH) ((**A**) F_(8,18)_ = 23.55, *p* < 0.0001, R^2^ = 0.9128) and DJ-1/GAPDH ((**B**) F_(8,18)_ = 14.52, *p* < 0.0001, R^2^ = 0.8658) protein expression were assessed by Western blotting. Original uncut Western blot images are shown in Supplementary Figure S8. The data are expressed as mean ± SEM with n = 3 and analyzed by one-way analysis of variance (ANOVA) followed by Tukey’s post hoc test. * *p* < 0.05, ** *p* < 0.01, and *** *p* < 0.001 significantly different from control cells. (**C**) Immunofluorescence staining of cells with tyrosine hydroxylase antibody (red) and DAPI nuclear stain (blue). Representative images from three independent experiments are shown as 40× magnification (scale bar = 100 μm). Separate image panels are shown in Supplementary Figure S4.

The levels of the rate-limiting enzyme for the biosynthesis of dopamine, tyrosine hydroxylase (TH), were significantly decreased (*p* < 0.001) by ROT at 0.5 and 1 μM (Figure 3A). However, ROA exposure for 48 h led to a significant decrease at 4 μM (*p* < 0.01) and 5 μM (*p* < 0.001). The protein deglycase (DJ-1; PARK7) protein, involved in various cellular processes and downregulating α-syn aggregate formation, is vulnerable to oxidative stress [24]. In this study, the level of DJ-1 was significantly decreased at ROT concentrations of 0.5 and 1 μM (*p* < 0.001) but only at a ROA concentration of 5 μM (*p* < 0.05) compared with the control cells (Figure 3B). These results reveal that ROA exerts milder neurotoxic effects compared to ROT in SH-SY5Y cells. In addition, the fluorescence signal of TH was detected in SH-SY5Y cells (Figure 3C and Supplementary Figure S4). The signals in ROT (0.5 and 1 μM) and ROA (3, 4, and 5 μM) are relatively weak compared to control which exhibited stronger red fluorescence of TH.

### 3.3. Effects of ROT or ROA on p-S129 and Total α-Syn Protein Expressions in SH-SY5Y Cells

**Figure 4:**
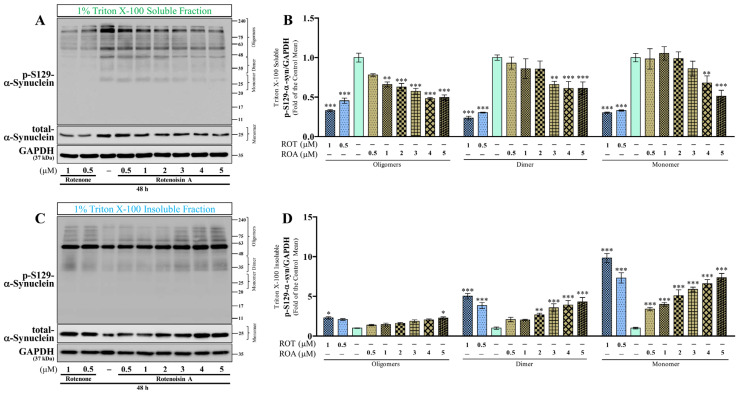
ROT or ROA on α-syn. Cells incubated with ROT or ROA for 48 h were collected and cell lysates were prepared as 1% triton X-100 soluble and insoluble (2% SDS soluble) fractions. p-Ser129 α-syn, total α-syn, and GAPDH were analyzed from 1% triton X-100 soluble (**A**) and insoluble (**C**) fractions by Western blotting. The bar graphs represent the ratios for oligomeric, dimeric, and monomeric p-Ser129 α-syn/GAPDH from triton X-100 soluble ((**B**) row factor F_(2,54)_ = 10.88, *p* = 0.0001; column factor F_(8,54)_ = 41.77, *p* < 0.0001; interaction F_(16,54)_ = 2.300; *p* = 0.0118) and insoluble ((**D**) row factor F_(2,54)_ = 265.1, *p* < 0.0001; column factor F_(8,54)_ = 48.36, *p* < 0.0001; interaction F_(16,54)_ = 9.466; *p* < 0.0001) fractions. Original uncut Western blot images are shown in Supplementary Figure S9. The data are expressed as mean ± SEM with n = 3 and analyzed by two-way ANOVA followed by Bonferroni post hoc test. * *p* < 0.05, ** *p* < 0.01, and *** *p* < 0.001 significantly different from control cells.

Phosphorylation at Serine 129 (p-S129) in α-syn increases abnormal accumulation of α-syn aggregates in the cytoplasm, promoting the progression of PD [25]. Western blot analyses of the triton X-100 soluble and insoluble lysate fractions were performed to detect the oligomeric, dimeric, and monomeric forms of p-S129 and monomeric forms of total α-syn protein expression levels based on their molecular weight (Figure 4 and Supplementary Figure S5). Western blotting of triton X-100 soluble fraction demonstrated that ROT toxicity for 48 h significantly decreased the levels of the oligomeric, dimeric, and monomeric forms of p-S129 α-syn/GAPDH (*p* < 0.001; Figure 4A,B), as well as the monomeric total α-syn/GAPDH form (*p* < 0.001 by 0.5 μM; *p* < 0.01 by 1 μM; Figure 4A and Supplementary Figure S5B). The levels of the oligomeric (*p* < 0.01 by 1 μM; *p* < 0.001 by 2, 3, 4, and 5 μM), dimeric (*p* < 0.01 by 3 μM; *p* < 0.001 by 4 and 5 μM), and monomeric (*p* < 0.01 by 4 μM; *p* < 0.001 by 5 μM) forms of p-S129 α-syn/GAPDH, and total α-syn/GAPDH (*p* < 0.05 by 1 μM; *p* < 0.01 by 2, 3, and 4 μM; *p* < 0.001 by 5 μM) were decreased by ROA toxicity (Figure 4A,B and Supplementary Figure S5B). Additionally, the soluble monomeric p-S129/total α-syn ratio was decreased by ROT (*p* < 0.01 by 0.5 μM; *p* < 0.001 by 1 μM; Figure 4A and Supplementary Figure S5A). However, ROA at 1 and 2 μM significantly increased (*p* < 0.01) the monomeric p-S129/total α-syn ratio (Supplementary Figure S5A).

Furthermore, the oligomeric (*p* < 0.05 by 1 μM of ROT and 5 μM of ROA), dimeric (*p* < 0.01 by 2 μM of ROA; *p* < 0.001 by 0.5 and 1 μM of ROT, and 3, 4, and 5 μM of ROA), and monomeric (*p* < 0.001 by ROT and ROA) p-S129 α-syn/GAPDH (Figure 4C,D), as well as the monomeric total α-syn/GAPDH (*p* < 0.05 by 0.5 μM ROT, and 3 and 4 μM of ROA; *p* < 0.01 by 5 μM of ROA; *p* < 0.001 by 1 μM of ROT; Supplementary Figure S5D), were increased in the triton X-100 insoluble fraction cell lysates. The increased levels of monomeric p-S129 and total α-syn subsequently resulted in an increased monomeric p-S129/total α-syn ratio (*p* < 0.001 by ROT; *p* < 0.05 by 1 μM; *p* < 0.01 by 2 μM; *p* < 0.001 by 3, 4, and 5 μM by ROA; Supplementary Figure S5C). In summary, our findings demonstrated that the soluble monomeric p-S129/total α-syn level increased in response to ROA but decreased in response to ROT compared to the control group (Supplementary Figure S5A), indicating that the neuropathological changes were more severe in the cells exposed to ROT compared to ROA.

### 3.4. Effects of ROT or ROA on Neuronal Marker Protein Expressions in SH-SY5Y Cells

**Figure 5:**
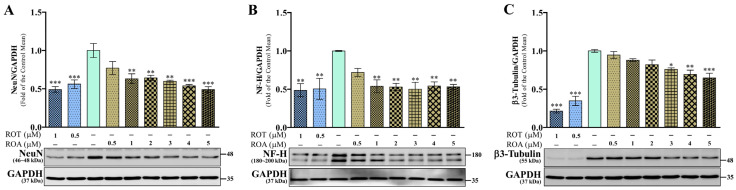
ROT or ROA on neuronal markers. Cells incubated with ROT or ROA for 48 h were collected, cell lysates were prepared, and the levels of NeuN/GAPDH ((**A**) F_(8,18)_ = 8.669, *p* < 0.0001, R^2^ = 0.7939), NF-H/GAPDH ((**B**) F_(8,18)_ = 4.961, *p* = 0.0023, R^2^ = 0.6880), and β3-Tubulin/GAPDH ((**C**) F_(8,18)_ = 36.19, *p* < 0.0001, R^2^ = 0.9415) protein expression were assessed by Western blotting. Original uncut Western blot images are shown in Supplementary Figure S10. The data are expressed as mean ± SEM with n = 3 and analyzed by one-way ANOVA followed by Tukey’s post hoc test. * *p* < 0.05, ** *p* < 0.01, and *** *p* < 0.001 significantly different from control cells.

Several neuronal proteins lose their functions after interacting with phosphorylated or oligomerized α-syn [16]. In this study, the protein expression of the neuronal markers neuronal nuclei (NeuN), neurofilament-heavy (NF-H), and β3-tubulin (Tuj1) was evaluated. As expected, the results showed a significant decrease in the expression levels of NeuN (*p* < 0.001; Figure 5A), NF-H (*p* < 0.01; Figure 5B), and β3-tubulin (*p* < 0.001; Figure 5C) in the ROT toxicity groups compared with the control group. Similarly, ROA toxicity also significantly decreased the NeuN (*p* < 0.01 by 1, 2, and 3 μM; *p* < 0.001 by 4 and 5 μM), NF-H (*p* < 0.01 by 1, 2, 3, 4, and 5 μM), and β3-tubulin (*p* < 0.05 by 3 μM; *p* < 0.01 by 4 μM; *p* < 0.001 by 5 μM) protein expressions, as shown in Figure 5A, Figure 5B, and Figure 5C, respectively.

### 3.5. Effects of ROT or ROA on Akt, ERK, and SAPK Protein Expressions in SH-SY5Y Cells

**Figure 6:**
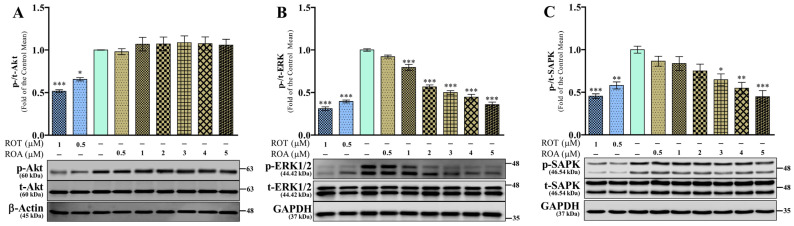
ROT or ROA on Akt and MAPKs. Cells incubated with ROT or ROA for 48 h were collected, cell lysates were prepared, and the levels of p-Akt (Ser473)/t-Akt ((**A**) F_(8,18)_ = 12.26, *p* < 0.0001, R^2^ = 0.8450), p-ERK (Thr202/Tyr204)/t-ERK ((**B**) F_(8,18)_ = 106.3, *p* < 0.0001, R^2^ = 0.9793), p-SAPK (Thr183/Tyr185)/t-SAPK ((**C**) F_(8,18)_ = 9.592, *p* < 0.0001, R^2^ = 0.8100), and β-actin or GAPDH were assessed by Western blotting. Original uncut Western blot images are shown in Supplementary Figure S11. The data are expressed as mean ± SEM with n = 3 and analyzed by one-way ANOVA followed by Tukey’s post hoc test. * *p* < 0.05, ** *p* < 0.01, and *** *p* < 0.001 significantly different from control cells.

Next, we analyzed the kinase signaling pathways by assessing the phosphorylation status of protein kinase B (Akt/PKB), extracellular signal-regulated kinase (ERK), and stress-activated protein kinase/Jun amino-terminal kinase (SAPK/JNK). ROT-induced toxicity reduced the phosphorylation of Akt at Ser473/t-Akt (*p* < 0.05 by 0.5 μM; *p* < 0.001 by 1 μM; Figure 6A), p-Akt/β-actin (*p* < 0.05 by 0.5 μM; *p* < 0.01 by 1 μM; Supplementary Figure S6A), p-ERK1/2 at Thr202 and Tyr204/t-ERK1/2 (*p* < 0.001; Figure 6B), p-ERK/GAPDH (*p* < 0.001; Supplementary Figure S6C), and p-SAPK at Thr183 and Tyr185/t-SAPK (*p* < 0.01 by 0.5 μM; *p* < 0.001 by 1 μM; Figure 6C), and p-SAPK/GAPDH (*p* < 0.01 by 0.5 μM; *p* < 0.001 by 1 μM; Supplementary Figure S6E). Moreover, ROA did not change the ratios of p-Akt/t-Akt and p-Akt/β-actin (Figure 6A and Supplementary Figure S6A) but dose-dependently decreased the p-ERK/t-ERK (*p* < 0.001 by 1, 2, 3, 4, and 5 μM; Figure 6B), p-ERK/GAPDH (*p* < 0.05 by 1 μM; *p* < 0.001 by 2, 3, 4, and 5 μM; Supplementary Figure S6C), p-SAPK/t-SAPK and p-SAPK/GAPDH (*p* < 0.05 by 3 μM; *p* < 0.01 by 4 μM; *p* < 0.001 by 5 μM; Figure 6C and Supplementary Figure S6E). The ratios of t-Akt/β-actin, t-ERK/GAPDH, and t-SAPK/GAPDH (Supplementary Figure S6B,D,F) were not affected by ROT or ROA in SH-SY5Y cells. These results suggest that ROT can significantly inhibit Akt and MAPKs signaling, but ROA differs in that it inhibits only MAPKs.

### 3.6. Effects of ROT or ROA on Apoptotic Protein Expressions in SH-SY5Y Cells

**Figure 7:**
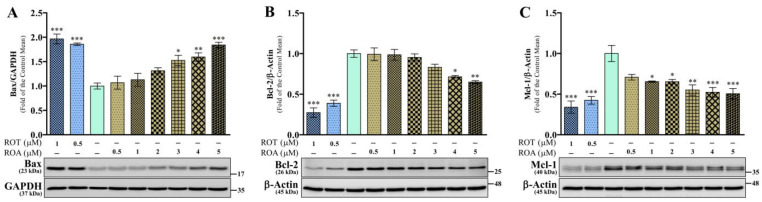
ROT or ROA on Bcl-2 family proteins. Cells incubated with ROT or ROA for 48 h were collected, cell lysates were prepared, and the levels of Bax/GAPDH ((**A**) F_(8,18)_ = 16.18, *p* < 0.0001, R^2^ = 0.8779), Bcl-2/β-actin ((**B**) F_(8,18)_ = 31.34, *p* < 0.0001, R^2^ = 0.9330), and Mcl-1/β-actin ((**C**) F_(8,18)_ = 10.53, *p* < 0.0001, R^2^ = 0.8239) were assessed by Western blotting. Original uncut Western blot images are shown in Supplementary Figure S12. The data are expressed as mean ± SEM with n = 3 and analyzed by one-way ANOVA followed by Tukey’s post hoc test. * *p* < 0.05, ** *p* < 0.01, and *** *p* < 0.001 significantly different from control cells.

The levels of proapoptotic bcl-2-like protein 4 (Bax), antiapoptotic b-cell lymphoma 2 (Bcl-2), and myeloid cell leukemia 1 (Mcl-1) protein expression were examined to assess ROT or ROA toxicity. ROT exposure for 48 h significantly increased the Bax/GAPDH (*p* < 0.001; Figure 7A), while decreasing Bcl-2/β-actin (*p* < 0.001; Figure 7B), and Mcl-1/β-actin (*p* < 0.001; Figure 7C) protein expression levels compared to the control group. ROA significantly increased Bax expression (*p* < 0.05 by 3 μM; *p* < 0.01 by 4 μM; *p* < 0.001 by 5 μM; Figure 7A) with inhibited levels of Bcl-2 (*p* < 0.05 by 4 μM, *p* < 0.01 by 5 μM; Figure 7B), and Mcl-1 (*p* < 0.05 by 1 and 2 μM, *p* < 0.01 by 3 μM, *p* < 0.001 by 4 and 5 μM; Figure 7C) in SH-SY5Y cells. Additionally, Bcl-2 can bind to Bax to form Bcl-2:Bax heterodimers, thereby attenuating the apoptotic effect of Bax. In this study, ROT or ROA caused an increase in the Bax/Bcl-2 ratio while reducing the Bcl-2/Bax ratio (Supplementary Figure S7A,B). These results show that the apoptotic-inducing effects of ROA on Bcl-2 family proteins are also milder compared to those of ROT.

**Figure 8:**
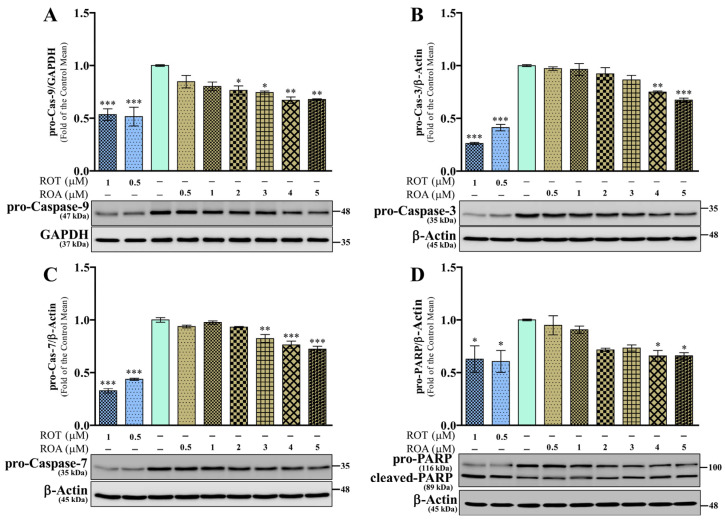
ROT or ROA on apoptosis markers. Cells incubated with ROT or ROA for 48 h were collected, cell lysates were prepared, and the levels of pro-Caspase-9/GAPDH ((**A**) F_(8,18)_ = 10.87, *p* < 0.0001, R^2^ = 0.8285), pro-Caspase-3/β-actin ((**B**) F_(8,18)_ = 60.88, *p* < 0.0001, R^2^ = 0.9644), pro-Caspase-7/β-actin ((**C**) F_(8,18)_ = 101.2, *p* < 0.0001, R^2^ = 0.9783), and pro-PARP/β-actin ((**D**) F_(8,18)_ = 4.923, *p* = 0.0024, R^2^ = 0.6863) were assessed by Western blotting. Original uncut Western blot images are shown in Supplementary Figure S13. The data are expressed as mean ± SEM with n = 3 and analyzed by one-way ANOVA followed by Tukey’s post hoc test. * *p* < 0.05, ** *p* < 0.01, and *** *p* < 0.001 significantly different from control cells.

As illustrated in Figure 8, the expressions of pro-Caspase-9/GAPDH (Figure 8A), pro-Caspase-3/β-actin (Figure 8B), and pro-Caspase-7/β-actin (Figure 8C) were significantly downregulated (*p* < 0.001) in the ROT toxicity group compared with the control group. ROA exposure significantly reduced the levels of pro-Cas-9 (*p* < 0.05 by 2 and 3 μM; *p* < 0.01 by 4 and 5 μM; Figure 8A), pro-Cas-3 (*p* < 0.01 by 4 μM; *p* < 0.001 by 5 μM; Figure 8B), and pro-Cas-7 (*p* < 0.01 by 3 μM; *p* < 0.001 by 4 and 5 μM; Figure 8C). Moreover, the pro-poly(ADP-ribose) polymerase (PARP)/β-actin level was markedly decreased (*p* < 0.05; Figure 8D) with increased levels of cleaved-PARP/β-actin (*p* < 0.05 and *p* < 0.001 by 0.5 and 1 μM, respectively; Supplementary Figure S7C), as well as an increase in the ratio of cleaved/pro-PARP (*p* < 0.001, Supplementary Figure S7D) by ROT toxicity. ROA decreased the level of pro-PARP/β-actin (*p* < 0.05 by 4 and 5 μM; Figure 8D) by increasing the level of cleaved-PARP/β-actin (*p* < 0.05 and *p* <0.001 by 4 and 5 μM, respectively; Supplementary Figure S7C), resulting in an increased ratio of cleaved/pro-PARP (*p* < 0.01 by 4 μM, *p* < 0.001 by 5 μM; Supplementary Figure S7D) in SH-SY5Y cells. These results demonstrate that ROT- and ROA- induced toxicity initiates apoptosis in SH-SY5Y cells.

### 3.7. Effects of ROT or ROA on Autophagy and BiP Protein Expressions in SH-SY5Y Cells

**Figure 9:**
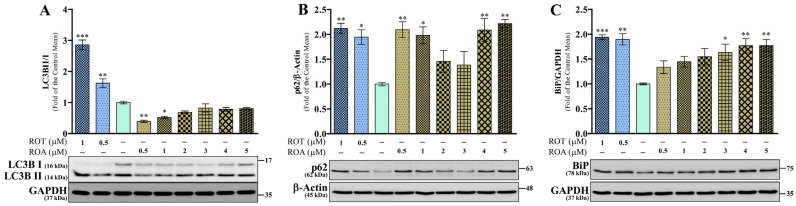
ROT or ROA on autophagy and ER. Cells incubated with ROT or ROA for 48 h were collected, cell lysates were prepared, and the levels of LC3BII/I ((**A**) F_(8,18)_ = 70.81, *p* < 0.0001, R^2^ = 0.9692), p62/β-actin ((**B**) F_(8,18)_ = 5.960, *p* = 0.0008, R^2^ = 0.7259), and BiP/GAPDH ((**C**) F_(8,18)_ = 6.047, *p* = 0.0008, R^2^ = 0.7288) were assessed by Western blotting. Original uncut Western blot images are shown in Supplementary Figure S14. The data are expressed as mean ± SEM with n = 3 and analyzed by one-way ANOVA followed by Tukey’s post hoc test. * *p* < 0.05, ** *p* < 0.01, and *** *p* < 0.001 significantly different from control cells.

Microtubule-associated proteins 1A/1B light chain 3B (LC3B) is considered a marker of autophagosome formation in the early stage of autophagy, and the number of increased autophagosomes is indicated by the LC3BII/LC3BI ratio [26]. The ratio of LC3BII/LC3BI (Figure 9A) at 48 h was increased by ROT (*p* < 0.01 by 0.5 μM; *p* < 0.001 by 1 μM); however, ROA decreased this ratio (*p* < 0.01 by 0.5 μM; *p* < 0.05 by 1 μM). These results are attributed to the differential inhibition of LC3BII or LC3BI by ROT or ROA. The levels of LC3BII/GAPDH (Supplementary Figure S7E) were significantly decreased by ROT (*p* < 0.05 by 0.5 μM), whereas lower concentrations of ROA highly decreased this ratio, with its inhibitory effects being less pronounced at higher concentrations (*p* < 0.001 by 0.5, 1, and 2 μM; *p* < 0.01 by 3 and 4 μM; *p* < 0.05 by 5 μM). The levels of LC3BI/GAPDH (Supplementary Figure S7F) were also significantly decreased by ROT (*p* < 0.001) and ROA (*p* < 0.05 by 1 μM; *p* < 0.001 by 2 and 3 μM; *p* < 0.01 by 4 and 5 μM).

Aggregated proteins linked to sequestosome-1 (p62; ubiquitin binding protein) interact with LC3B for their degradation in autophagy [27]. In this study, the levels of p62 (Figure 9B) were increased by ROT (*p* < 0.05 by 0.5 μM; *p* < 0.01 by 1 μM) and ROA (*p* < 0.01 by 0.5, 4, and 5 μM; *p* < 0.05 by 1 μM). However, ROA at 2 and 3 μM did not change the levels of p62 compared to the control cells. Our findings indicated that the increased LC3BII/I ratios and p62 levels by ROT-induced toxicity impaired the autophagic flux, whereas ROA induces the autophagic flux in lower doses (decreased LC3BII/I with increased p62) and blocks this flux when administered at higher doses (no changes in LC3BII/I with increased p62). These results demonstrate that the autophagy impairments on SH-SY5Y cells are dose-dependent.

The ER is an organelle involved in protein production and transport; however, the accumulation of misfolded proteins such as α-syn tends to increase during ER dysfunction [28]. In this study, the levels of binding immunoglobulin protein/78 kDa glucose-regulated protein (BiP/GRP78) (Figure 9C) were increased by the toxicity of both ROT (*p* < 0.001) or ROA (*p* < 0.05 by 3 μM; *p* < 0.01 by 4 and 5 μM). Lower doses of ROA showed a non-significant increase in the levels of BiP, suggesting concentration-dependent ER dysfunction.

## 4. Discussion

SH-SY5Y cells grown in DMEM with 1% FBS medium differentiated into neuron-like cells with neurite outgrowth (Supplementary Figure S2C) were used for the investigation of neurodevelopmental disorders. Another study also reported that reducing FBS to 1% induced neuronal differentiation, leading to a significant reduction in confluence (*p* < 0.001), with no significant differences under 1% FBS compared to 1% FBS + retinoic acid (10 μM) for 7 days [29]. ROT treatment results in neurotoxicity in SH-SY5Y cells [16,17,18], which indicate that ROT is toxic to normal cells, prompting studies focused on structural modifications of ROT to minimize neurotoxicity and environmental contamination, in addition to enhancing bioactivity. The chemical transformation on the C-12 ketone moiety of ROT, modified to an epoxy ring of ROA, was evaluated for properties related to inducing neurotoxicity.

In this study, ROT exposure for 48 h markedly diminished cell viability and altered cell morphology in SH-SY5Y cells with increasing concentrations. However, ROA treatment showed less toxicity compared to the same concentrations of ROT in SH-SY5Y cells during 48 h. In a previous report, ROA was weaker than ROT in the human breast cancer MCF-7 cells or primary epidermal keratinocytes HEKa cells [23]. Therefore, the derivative ROA has a lower cytotoxic effect after structural changes compared to its parent compound ROT.

Tyrosine hydroxylase (TH) is the rate-limiting enzyme responsible for the conversion of tyrosine into DOPA in the catecholamine biosynthesis pathway in physiological brain functions [16]. ROT (0.5 and 1 μM)-induced toxicity reduced TH activity, whereas ROA at 4 and 5 μM reduced the expression of TH. The observed reduction in TH by ROT and ROA suggests that these compounds impairing dopamine synthesis and inducing synaptic dysfunction. DJ-1 present in synaptic terminals, acts as a sensor of oxidative stress [30] controls protein synthesis by amending multiple transcription factors [31] and can inhibit α-syn aggregation at an early step in the process [24]. In this study, DJ-1 protein expression decreased in response to ROT exposure, whereas ROA at 5 μM only induced a moderate inhibition of DJ-1 levels.

In turn, a recent related study reported that DJ-1 deficiency may aggravate the accumulation of α-syn [24]. α-syn can occur as membrane-bound or cytosolic forms, playing a physiological role in membrane-membrane interactions at the pre-synapse and acting as the driving force of aggregate formation [32]. PD is also associated with the formation of misfolded α-syn aggregates, the main component in Lewy bodies (LBs). α-syn is phosphorylated at Serine 129 [25]. The oligomeric p-S129 α-syn can reflect the pathogenesis of PD and is used as a potential biomarker for PD [33].

In this study, the oligomeric, dimeric, and monomeric p-S129 α-syn, or monomeric total α-syn, and the ratio of monomeric p-S129/total α-syn protein expressions in the triton X-100 insoluble cell fraction in SH-SY5Y cells may be influenced by increased toxicity induced by ROT and ROA. Presumably, ROT appears to facilitate the phosphorylation of α-syn at S129, leading to aggregate formation with impaired normal cellular function, which results in more severe neuronal cell loss compared to the effects of ROA. The results also showed that the proportion of triton X-100 soluble p-S129/t-α-syn monomer at 1 and 2 μM of ROA was significantly higher than that of normal cells, whereas ROT exerted inhibitory effects. Therefore, additional studies are needed to explore the mechanisms that drive the differential toxicity of ROA.

In addition to TH, other specific proteins are present in neurons. Neuronal nuclear protein (NeuN), used as a marker of postmitotic neurons, is associated with neuronal differentiation [34]. Neurofilaments (NFs) are intermediate filament proteins that are important for axonal growth and neural transmission [35]. Neurofilament heavy chain (NF-H) varies in size, with 180 to 200 kDa subunits, and is an extremely long-lived protein involved in the three-dimensional structure of axoplasm [36]. β3-tubulin (Tuj1) is a specific protein serving in the organization of cytoskeleton proteins required for neurite outgrowth. In this study, both ROT- and ROA-induced neurotoxicity led to the decreased expression of these proteins in SH-SY5Y cells. These analyses suggest that ROT and ROA affect the progression of axonal injury involving disruption of neurofilament organization, cytoskeleton dysfunction, and impairment of axonal transport.

MAPKs and Akt/PKB are protein kinases that participate in signaling pathways involved in various cellular functions, including cell survival, cell cycle progression, differentiation, development, and apoptosis [37,38]. In this study, we observed that ERK1/2 is phosphorylated as Thr202/Tyr204 (p42/p44), SAPK/JNK is phosphorylated as Thr183/Tyr185 (p46/p54), and p-Akt is phosphorylated as Ser473 (62 kDa) in response to diverse extracellular stimuli. Both ROT and ROA reduced the phosphorylation of ERK and SAPK, but only ROT reduced the phosphorylation of Akt.

DJ-1 is known to regulate ERK and Akt signaling pathways [31], with Akt being downstream of DJ-1 [39]. ROT toxicity induced a significant decrease in p-Akt and DJ-1 in this study. In turn, decreased p-Akt contributes to the loss of dopaminergic neurons in PD patients [40]. Moreover, the inhibition of both the Akt and ERK pathways is required to completely block the stimulation of mTORC1 signaling [41]. Collectively, our findings revealed that ROA-induced inhibition of phosphorylated ERK1/2 and SAPK was not associated with Akt phosphorylation. The differential effects of ROA remain somewhat unclear but may be attributable to structural transformations. These findings highlight the importance of studying the effects of chemical modifications of toxic compounds on their toxicity.

Mitochondria-mediated apoptotic cell death is responsible for neuronal loss in PD [42]. Bcl-2 family proteins possess either proapoptotic or antiapoptotic properties [43]. Bax integrates into the outer mitochondrial membrane, where Bcl-2 sequesters Bax, preventing their activation [44]. In this study, the expression of Bax was negatively correlated with Bcl-2 and Mcl-1 expressions during ROT or ROA toxicity. Additionally, the ratio between Bax and Bcl-2 used as a hallmark of mitochondrial permeability and a predictor of apoptosis [45,46]. According to our study, ROT and ROA caused a significant increase in the Bax/Bcl-2 ratio (decreased Bcl-2/Bax ratio) in SH-SY5Y cells that is associated with apoptosis induction.

Caspases are apoptosis-associated proteins initially synthesized as inactive pro-caspases whose cleaved products lead to their activation, promoting cell apoptosis [47,48]. The upregulation of Bax acts on ion channels on the mitochondrial membrane, causing pores in the membrane to open, thus allowing cytochrome-c to cross and be released into the cytoplasm in response to ROT exposure [42]. In turn, this initiates apoptosis through the activation of Cas-9 [49] further activate Cas-3/7 leading to biochemical and morphological changes [50]. Therefore, our data also demonstrated that decreased pro-Cas-9, -3, and -7 by ROT or ROA evidenced mitochondria-mediated apoptotic process becomes irreversible [50], leading to the induction of the formation of a cleaved 89 kDa PARP fragment detected in SH-SY5Y cells. Furthermore, a previous study reported that increased apoptosis by ROT and ROA was confirmed by Annexin V/propidium iodide staining in MCF-7 cells [23]. Overall, our data demonstrated that Bcl-2 family signaling pathways are involved in ROT-induced apoptosis and, to a lesser extent, in ROA-induced toxicity.

Autophagy is a degradation pathway regarded as a survival mechanism that recycles cytoplasm and other organelles within double membrane vesicles fused with lysosomes in response to various stimuli [42]. Autophagy can either promote or inhibit apoptosis. LC3B is expressed as LC3BI, which is conjugated with phosphatidylethanolamine into LC3BII conversion and then utilized for autophagosome formation [51]. p62 is involved in protein aggregate clearance via both autophagy and proteasome pathways [27]. After 48 h, both LC3BII and p62 upregulation became more significant in ROT-induced toxicity, indicating defective autophagy. ROT is reported to decrease the autophagy process, inducing neuronal degeneration with an increase in α-syn expression [52,53]. However, ROA-mediated regulation of LC3BI, II, and p62 varied in response to oxidative stress. Studies have reported that the levels of p62 increase when autophagy activation is required, whereas they decrease when autophagy is abundantly activated [54,55]. Our findings suggested that ROA induces protective autophagy at lower doses while inducing apoptosis with insufficient autophagic activity at higher doses, thus highlighting the importance of dose-dependent effects. Further studies involving additional autophagic markers would enhance our understanding of these relationships.

BiP/GRP78 is a member of the heat shock protein family and primarily acts as a protective molecule in the endoplasmic reticulum (ER) [56]. The expression of BiP on the cell surface facilitates the folding and assembly of newly synthesized proteins, correlating with unfolded protein response (UPR) signaling in normal cells [57]. BiP regulates ER stress proteins, including PERK, IRE1α, and ATF6 [58]. Our findings demonstrated that BiP protein expression was increased by ROT and higher doses of ROA in SH-SY5Y cells. α-syn aggregation is known to trigger ER stress via interaction with BiP in the ER lumen, initiating a UPR response [28]. This suggests that excessive stimuli by ROT or ROA may cause ER stress, activating pathways related to apoptosis and autophagy-related cell death.

## 5. Conclusions

**Figure 10:**
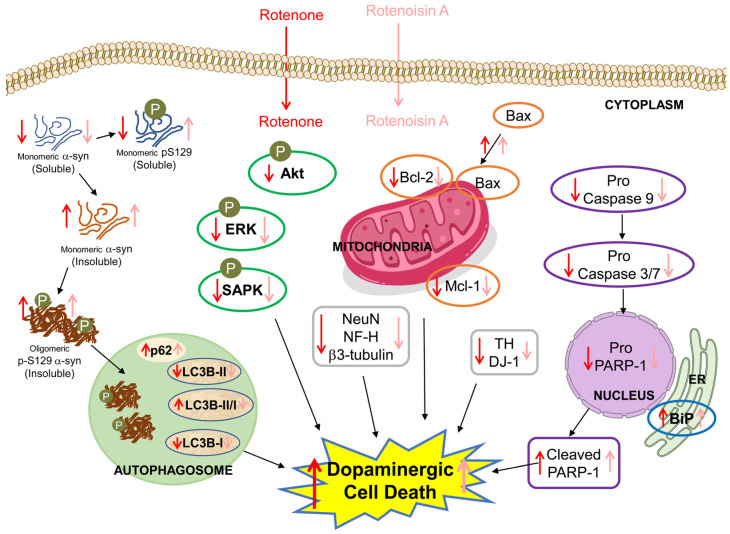
Diagrammatic representation of the various cellular protein signaling by toxicity of ROT and ROA. The downward arrows (↓) denote inhibitions, and the upward arrows (↑) denote stimulation. The color density indicates the level of toxicity; hence, dark red (ROT) is more toxic than light red (ROA) at the same concentration. Akt was phosphorylated by ROT, not ROA. The level of soluble monomeric p-S129 α-syn was inhibited by ROT, while ROA stimulated it. More interestingly, ROT increased the LC3B-II/I ratio, whereas this was downregulated by ROA.

ROT has been associated with toxicity to normal cells, particularly linked to neurodegenerative diseases. The structural modification of ketone moiety into epoxy ring at the C-12 position of the central dihydro-γ-pyrone ring of ROT generates its derivate ROA. These compounds have been reported for their anticancer effects. In this study, we examined the toxic effects induced by ROT and ROA in SH-SY5Y neuroblastoma cells. The comparison revealed different mechanisms of action involving oxidative stress, α-synuclein aggregation, neuronal-specific proteins, protein kinases, mitochondrial apoptosis, and autophagy (Figure 10). In general, ROA was shown to induce oxidative stress in neuronal cells. Upon comparing the effects produced by both compounds, ROT exhibited greater toxicity as a model for inducing Parkinson’s disease. The structural modification of ROA resulted in decreased toxicity compared to its parent compound ROT when reproduce in vitro cellular model of Parkinson’s disease. In addition to the above points, this finding suggests that structural modifications of highly toxic compounds could be a promising strategy for developing therapeutic anticancer candidates with reduced adverse effects on normal cells.

## Data Availability

All the data needed to assess the conclusions are exhibited in the article and Supplementary Materials; further inquiries can be directed to the corresponding author/s.

## Supplementary Materials

The following supporting information can be downloaded at: https://www.mdpi.com/article/10.3390/biomedicines12081703/s1, the supplementary PDF file contains supplementary Figures S1–S7, Table S1, and unedited chemiluminescence Figures S8–S14 mentioned in the main article.
