# Supplementary material for: Rotenone and Its Derivative, Rotenoisin A, Induce Neurodegeneration Differentially in SH-SY5Y Cells
Source: Biomedicines. 2024 Jul 31;12(8):1703. doi: 10.3390/biomedicines12081703 (PMC11351421; doi:10.3390/biomedicines12081703)
Supplement: Supplementary file 1 [file biomedicines-12-01703-s001.zip › biomedicines-3100440-supplementary.pdf]

Supplementary materials to

Article

Rotenone and its Derivative, Rotenoisin A, Induce Neurodegeneration Differentially in SH-SY5Y Cells

Mahesh Ramalingam [1,*,†], Sujeong Jang [1,†], Seongryul Kim [1], Hyoungwoo Bai [2,3], Gyeonghan Jeong [2], Byeong C. Kim [4,5] and Han-Seong Jeong [1,*]

1 Department of Physiology, Chonnam National University Medical School, Hwasun 58128, Republic of Korea; sujeong.jjang@gmail.com (S.J.); ryul5204@hanmail.net (S.K.)
2 Department of Radiation Science, Korea Atomic Energy Research Institute (KAERI), Jeongeup 56212, Republic of Korea; hbai@kaeri.re.kr (H.B); jkh4598@kaeri.re.kr (G.J)
3 Radiation Biotechnology and Applied Radioisotope Science, University of Science and Technology (UST), Daejeon 34113, Republic of Korea
4 Department of Neurology, Chonnam National University Medical School, Gwangju, 61469, Republic of Korea; byeong.kim7@gmail.com (B.C.K)
5 Department of Neurology, Chonnam National University Hospital, Gwangju, 61469, Republic of Korea
* Correspondence: ramalingammahesh@jnu.ac.kr (M.R.); jhsjeong@hanmail.net (H.-S.J.)
† These authors contributed equally to this work.

A **γ-Irradiation and high-performance liquid chromatography (HPLC):**

Rotenoisin A was prepared using the method described in our previous reports[21,23]. Briefly, a sample solution of rotenone (0.5 g) in MeOH (200 mL) in capped vials was exposed to 50 kGy (absorbed dose) of radiation. Irradiation was carried out at ambient temperature using a cobalt-60 irradiator (point source AECL, IR-79, MDS Nordion International Co. Ltd, Ottawa, ON, Canada) at the Advanced Radiation Technology Institute, Korea Atomic Energy Research Institute (Jeongup, Korea). The source strength was ~320 kCi, with a dose rate of 10 kGy h^{-1} at the location of the sample. The irradiated methanolic solution was immediately evaporated to remove the solvent and lyophilized. The dried irradiated solution was directly subjected to column chromatography over a silica gel column (2.5 cm i.d. × 32 cm) with $CHCl_3$-MeOH to yield pure rotenoisin A (129.1 mg).

High performance liquid chromatography (HPLC; LC-20AD system, Shimadzu, Tokyo, Japan) equipped with a photodiode array detector (PDA, SPD-M20A, Shimadzu) and a series of YMC-Pack ODS A-302 column (4.6 mm i.d. × 150 mm, particle size 5 µm; YMC Co., Kyoto, Japan) using an elution gradient of 20% to 100% MeCN in 0.1% HCOOH (detection: 280 nm; flow rate: 1.0 mL/min; oven temperature: 40 °C) were used to analyze the compounds.

B

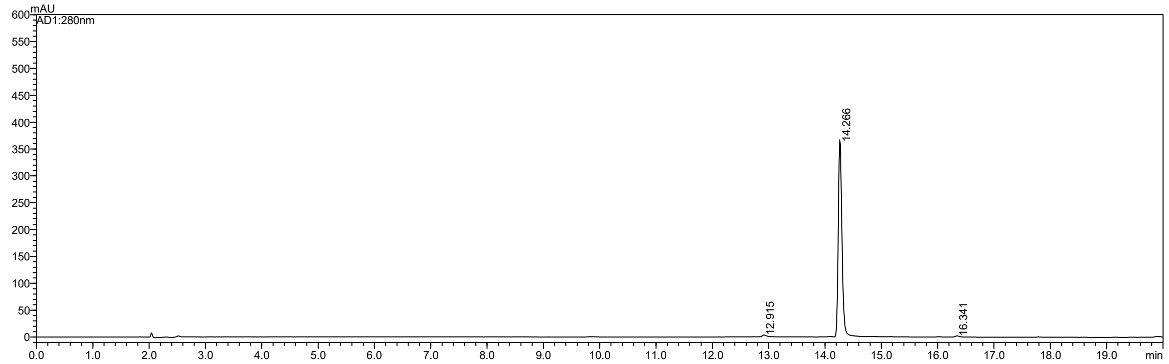

ID#	Ret.Time	Area	Height	Area%
**1**	12.915	25426	3296	1.4616
**2 (Rotenone)**	14.266	1700036	316833	97.7247
**3**	16.341	14155	1796	0.8137

C

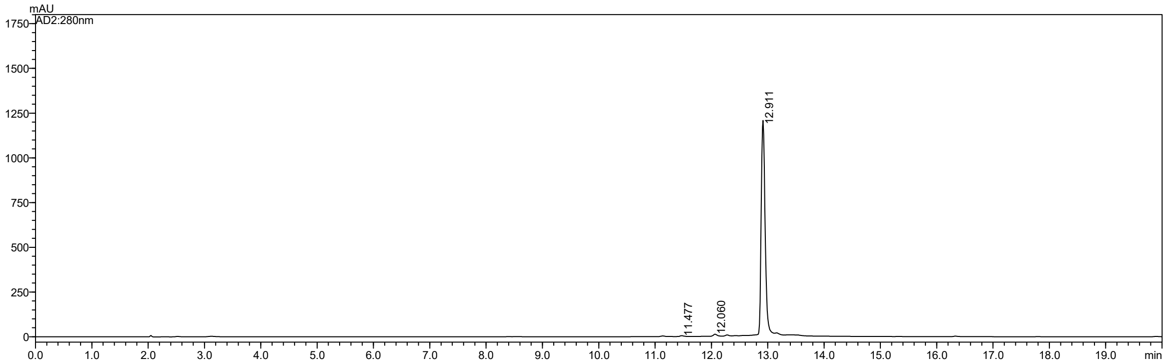

ID#	Ret.Time	Area	Height	Area%
**1**	11.477	20204	3704	0.3191
**2**	12.060	49948	10035	0.7889
**3 (Rotenoisin A)**	12.911	6261316	1126951	98.8920

Supplementary Figure S1. The processes of gamma irradiation, purification, and chromatography analysis of ROA (A) and HPLC chromatograms of ROT (B) and ROA (C).

References:

21. Park, C.H.; Chung, B.Y.; Lee, S.S.; Bai, H.W.; Cho, J.Y.; Jo, C.; Kim, T.H. Radiolytic transformation of rotenone with potential anti-adipogenic activity. *Bioorg. Med. Chem. Lett.* **2013**, *23*, 1099-1103, doi:10.1016/j.bmcl.2012.12.003.

23. Bak, D.H.; Kang, S.H.; Park, C.H.; Chung, B.Y.; Bai, H.W. A novel radiolytic rotenone derivative, rotenoisin A, displays potent anticarcinogenic activity in breast cancer cells. *J. Radiat. Res.* **2021**, *62*, 249-258, doi:10.1093/jrr/rrab005.

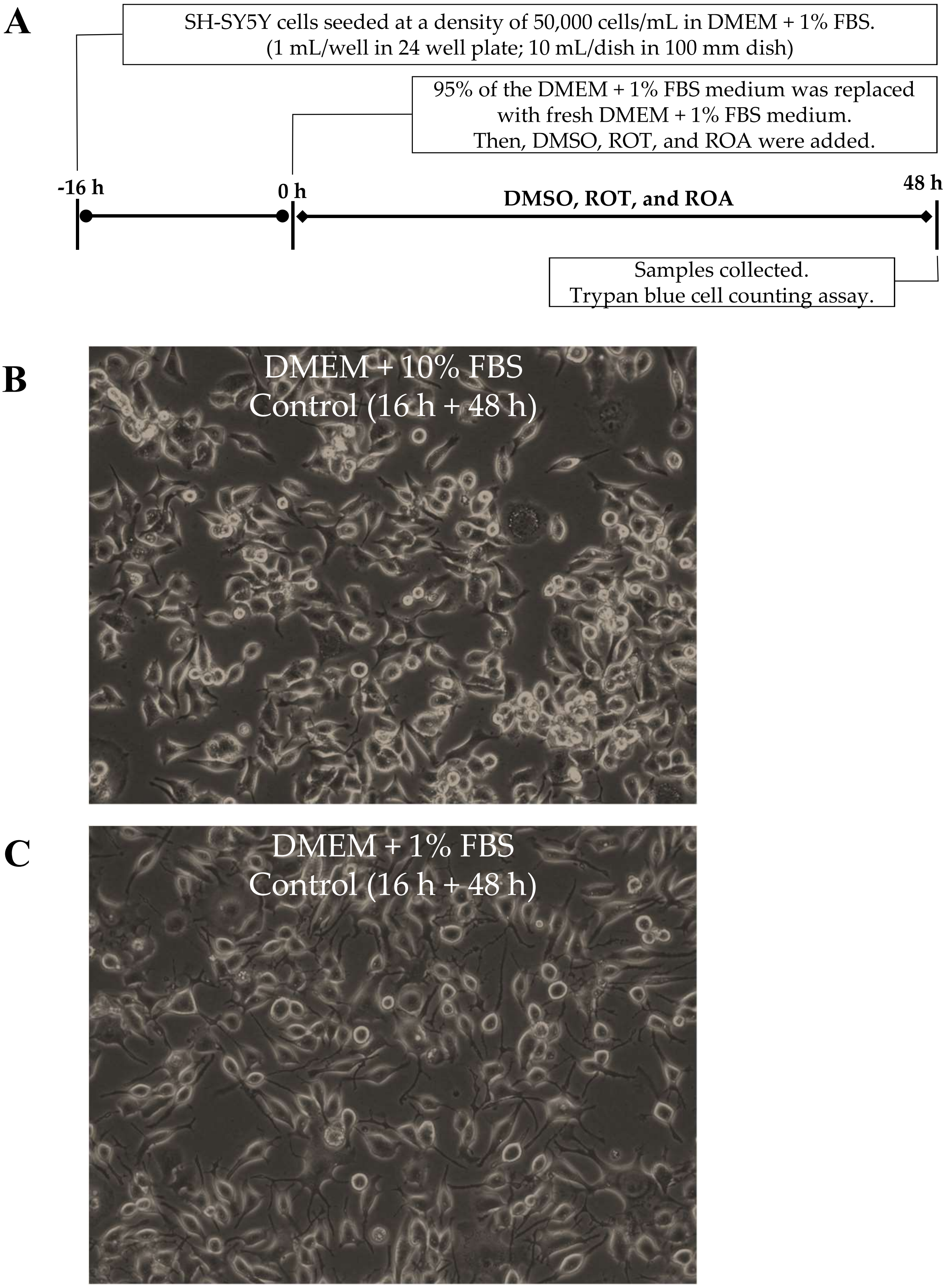

Supplementary Figure S2: The experimental study plan for various concentrations of ROT- and ROA-induced toxicity (A). SH-SY5Y cells grown with DMEM containing 10% FBS were re-seeded as 50,000 cells/ml in DMEM + 10% FBS and incubated for overnight (~16 h). 95% of the medium was replaced with fresh DMEM + 10% FBS medium and incubated further for 48 h (B). SH-SY5Y cells grown with DMEM containing 10% FBS were re-seeded as 50,000 cells/ml of DMEM + 1% FBS and incubated for overnight (~16 h). 95% of medium replaced with fresh DMEM + 1% FBS medium and incubated further for 48 h (C). Each picture is representative of three independent experiments.

D

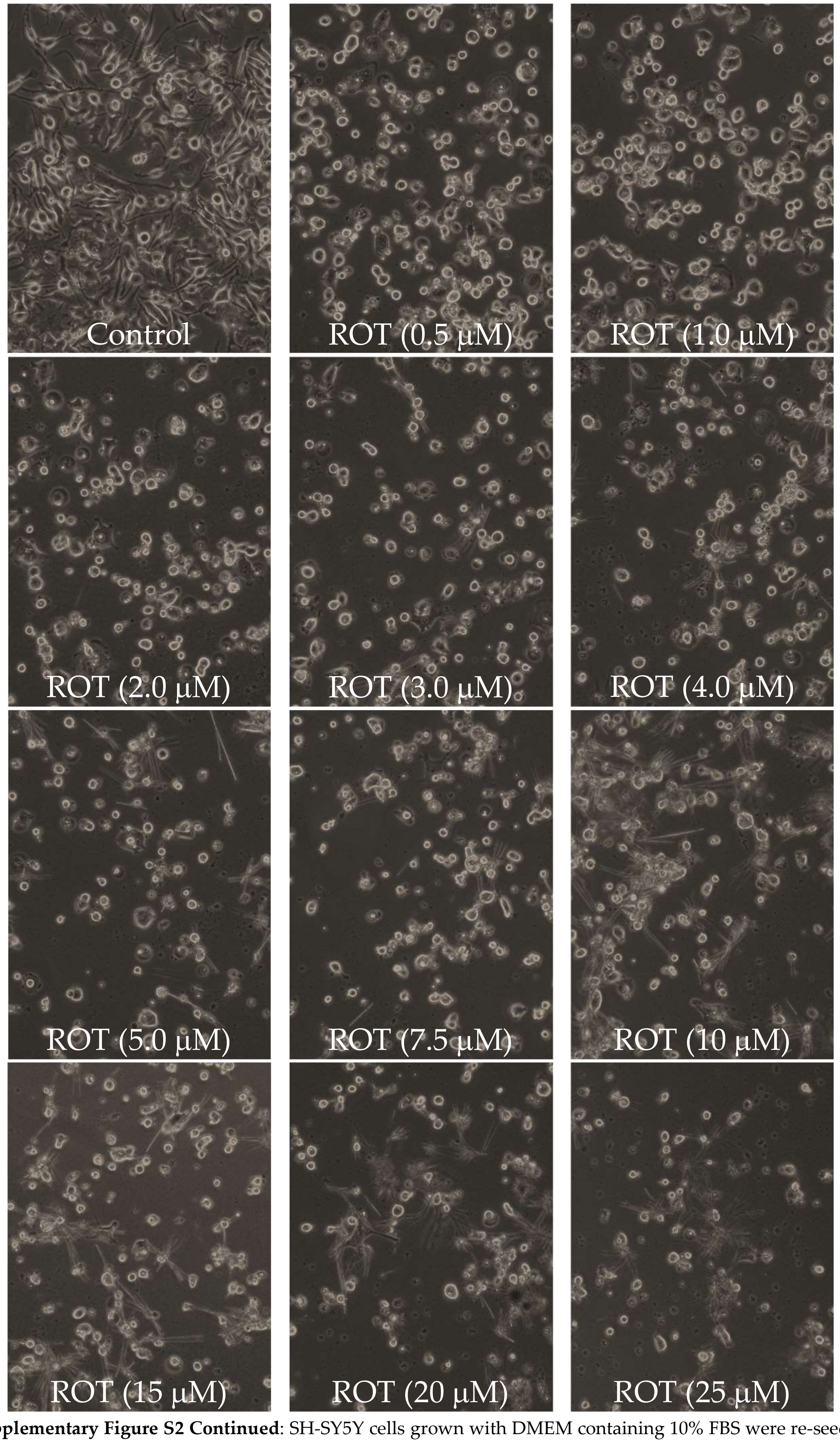

Supplementary Figure S2 Continued: SH-SY5Y cells grown with DMEM containing 10% FBS were re-seeded as 50,000 cells/ml of DMEM + 1% FBS and incubated for overnight (~16 h). 95% of the medium was replaced with fresh DMEM + 1% FBS medium, then, DMSO or various concentrations of ROT were added and incubated further for 48 h (D). Each picture is representative of three independent experiments.

E

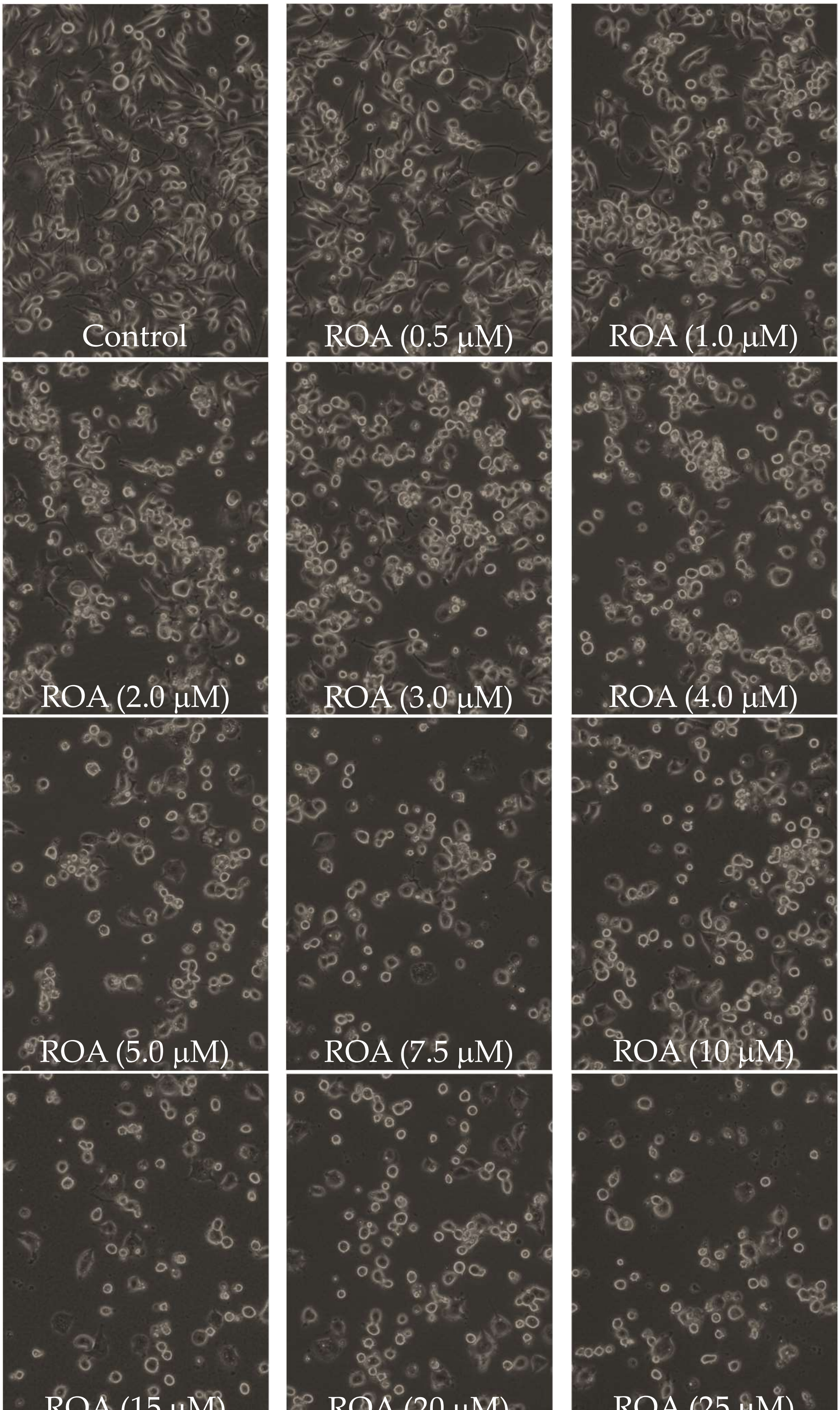

Supplementary Figure S2 Continued: SH-SY5Y cells grown with DMEM containing 10% FBS were re-seeded as 50,000 cells/ml of DMEM + 1% FBS and incubated for overnight (~16 h). 95% of the medium was replaced with fresh DMEM + 1% FBS medium, then, DMSO or various concentrations of ROA were added and incubated further for 48 h (E). Each picture is representative of three independent experiments.

A

(μM)	1	0.5	0.0	0.5	1	2	3	4	5
	**Rotenone**			**Rotenoisin A**					

48 h

B

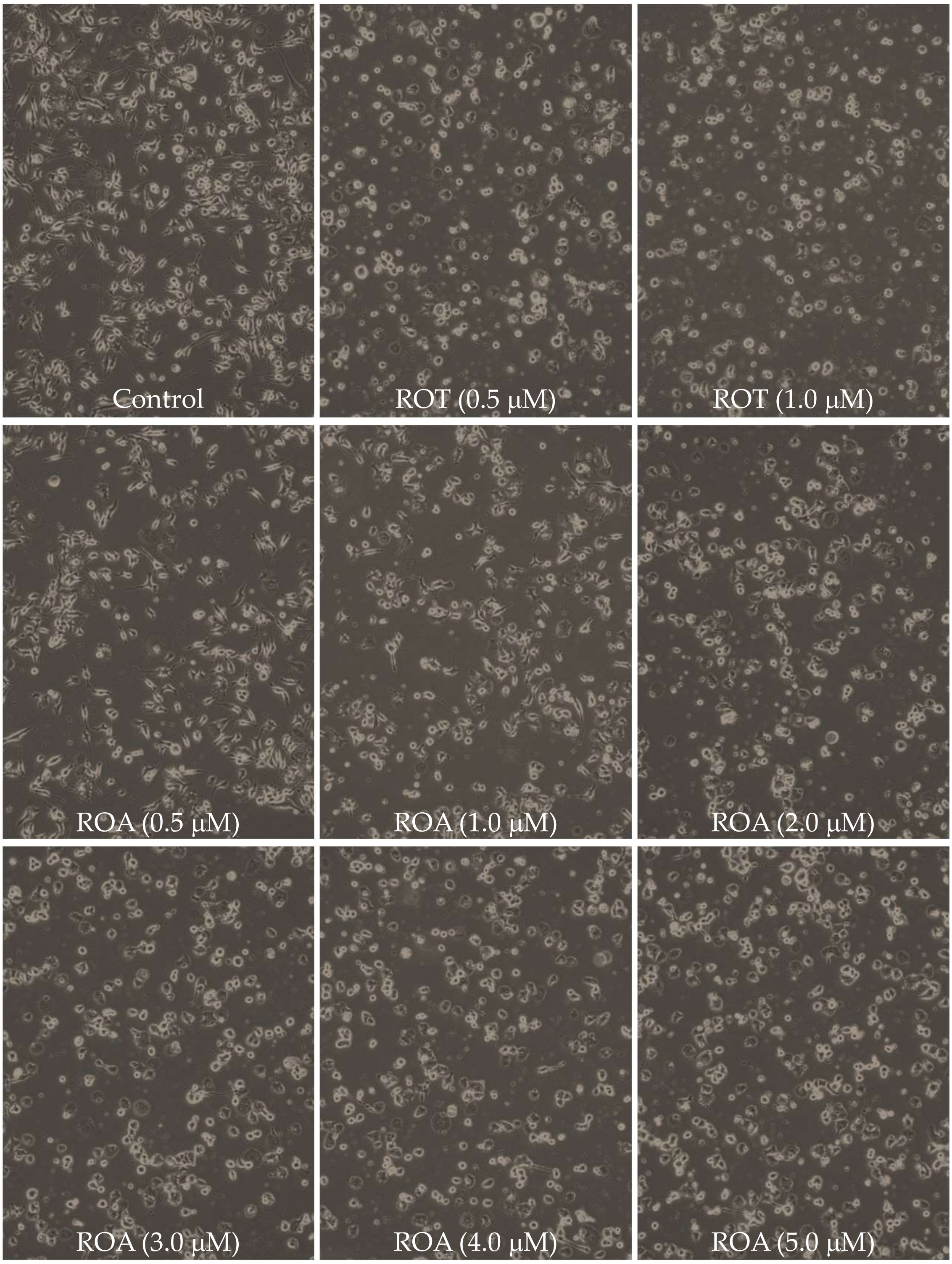

Supplementary Figure S3. (A) Western blotting experimental study plan with various concentrations of ROT- or ROA-induced toxicity for 48 h. (B) SH-SY5Y cells were seeded at a density of 50,000 cells/mL in DMEM + 1% FBS and incubated for overnight (~16 h). 95% of the medium was replaced with fresh DMEM + 1% FBS medium, then, DMSO, ROT, and ROA were added and incubated further for 48 h. Each picture is representative of three independent experiments.

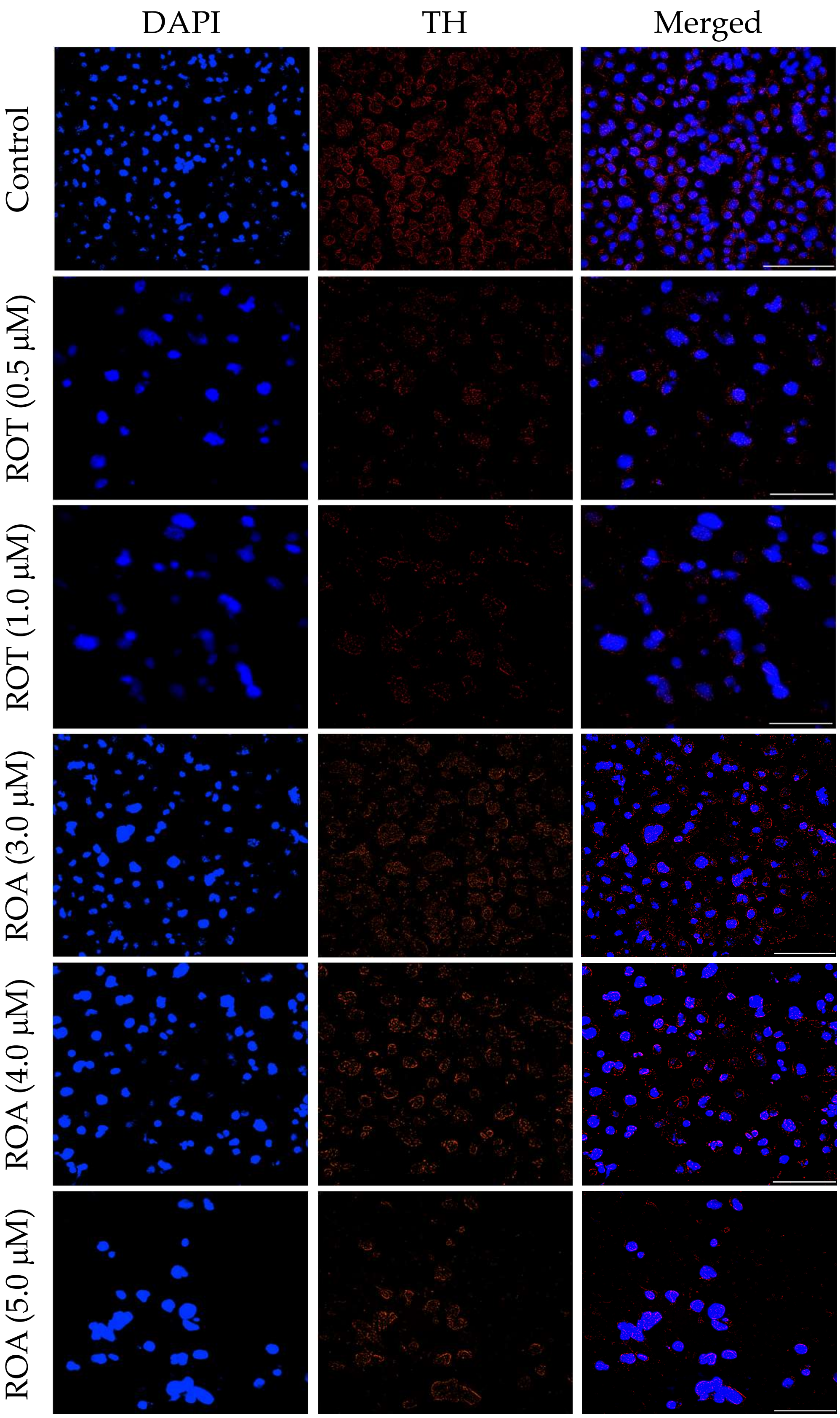

Supplementary Figure S4. SH-SY5Y cells were seeded at a density of 50,000 cells/ml in DMEM containing 1% FBS and incubated for overnight (~16 h). 95% of the medium was replaced with fresh DMEM + 1% FBS medium, then, DMSO, ROT, and ROA were added and incubated further for 48 h. Floating and adherent cells were harvested with cell culture medium. Then, cells were washed with PBS and immunofluorescence staining with tyrosine hydroxylase (red) and DAPI nuclear stain (blue). Overlay images were merged and shown as 40× magnification (scale bar = 100 µm). Each picture is representative of three independent experiments.

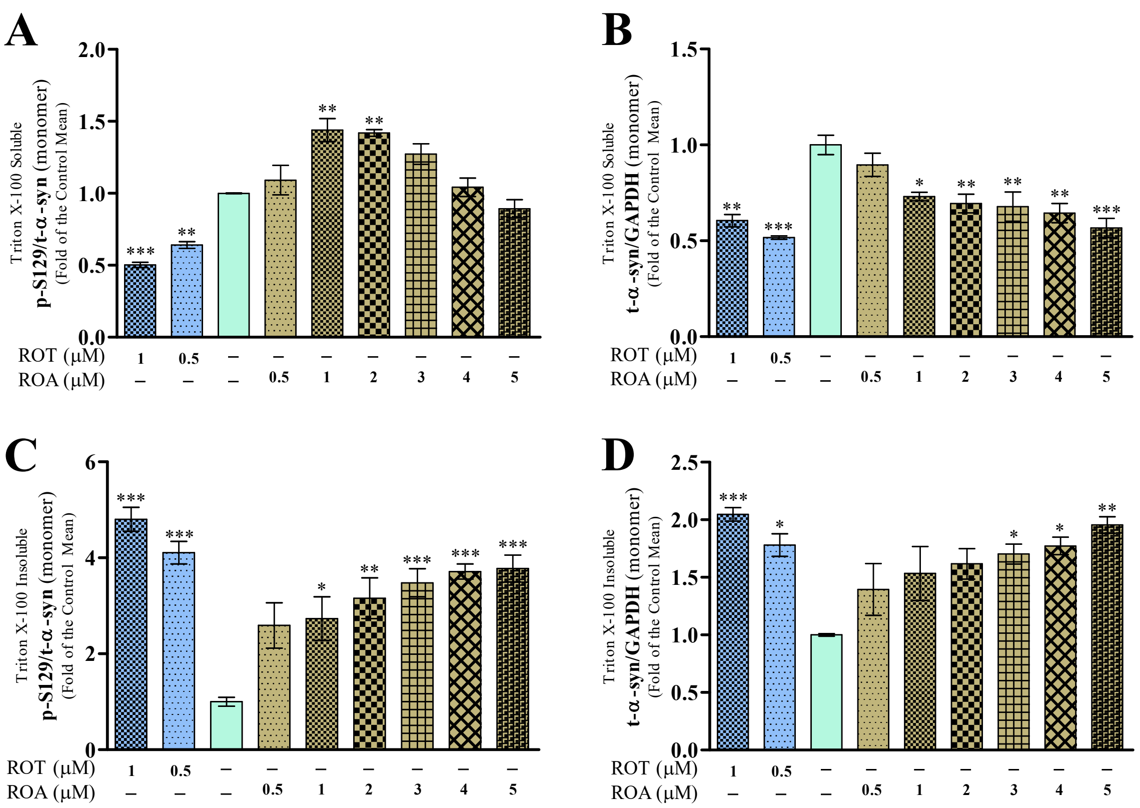

Supplementary Figure S5. SH-SY5Y cells were seeded at a density of 50,000 cells/ml in DMEM containing 1% FBS and incubated for overnight (~16 h). 95% of the medium was replaced with fresh DMEM + 1% FBS medium, then, DMSO, ROT, and ROA were added and incubated further for 48 h. Floating cells in cell culture medium were harvested and combined with adherent cells. Then, cells were washed with PBS and cell lysates were prepared as 1% Triton X-100 soluble and insoluble (2% SDS soluble) fractions. p-S129 α-syn, total α-syn, and GAPDH were analyzed from 1% Triton X-100 soluble (A,B) and insoluble (C,D) fractions by Western blotting. The bar graphs depict the ratio of soluble monomeric p-Ser129/total α-syn (A; $F_{(8,18)} = 29.72$, $p < 0.0001$, $R^2 = 0.9296$), soluble monomeric total α-syn/GAPDH (B; $F_{(8,18)} = 10.35$, $p < 0.0001$, $R^2 = 0.8214$), insoluble monomeric p-Ser129/total α-syn (C; $F_{(8,18)} = 11.49$, $p < 0.0001$, $R^2 = 0.8363$), and insoluble monomeric total α-syn/GAPDH (D; $F_{(8,18)} = 5.728$, $p = 0.0010$, $R^2 = 0.7180$). Data are expressed as mean ± SEM with n=3 and analyzed by one-way analysis of variance (ANOVA) followed by Tukey's *post hoc* test. *p<0.05, **p<0.01, and ***p<0.001 significantly different from control cells.

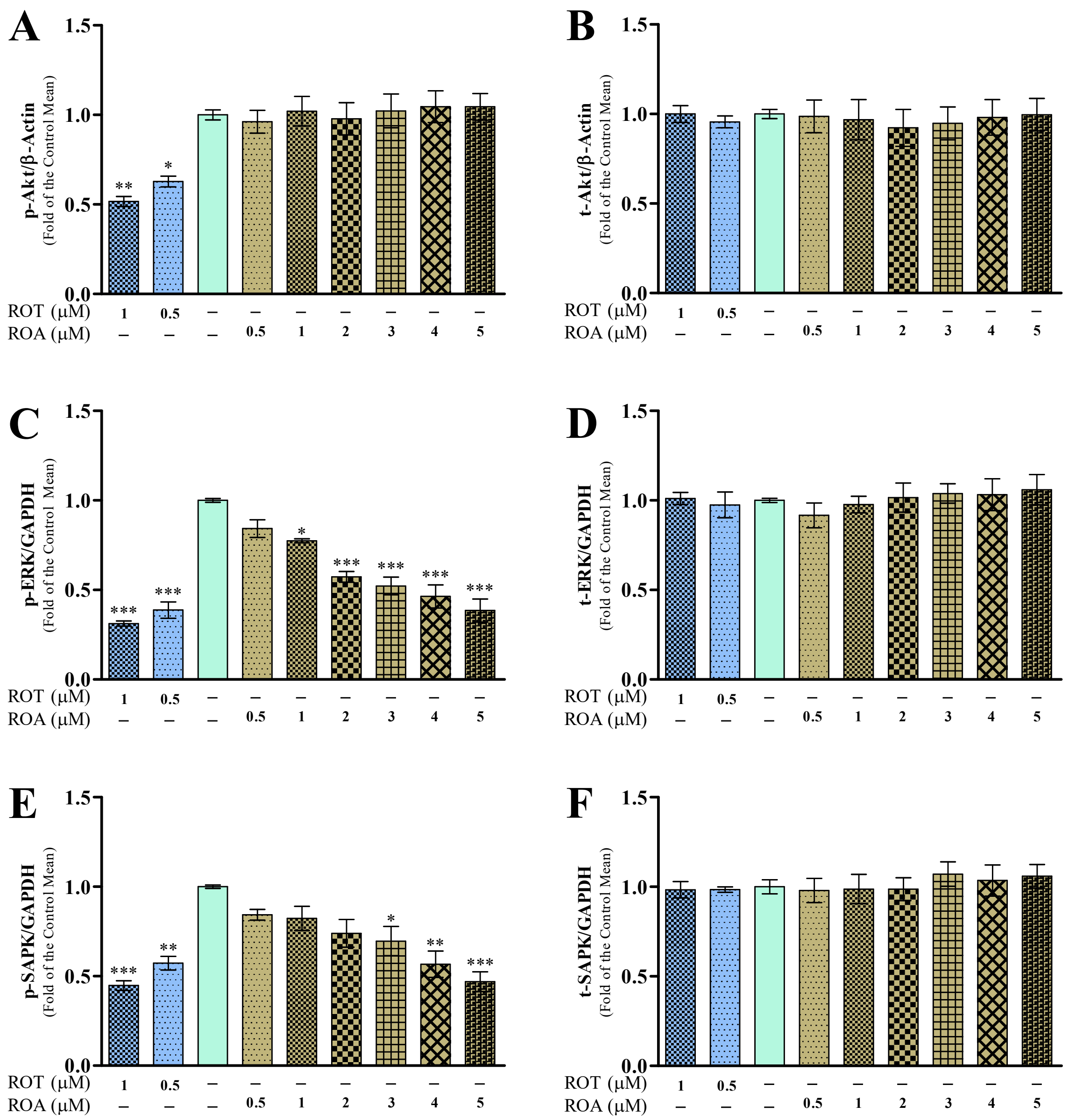

Supplementary Figure S6. SH-SY5Y cells were seeded at a density of 50,000 cells/ml in DMEM containing 1% FBS and incubated for overnight (~16 h). 95% of the medium was replaced with fresh DMEM + 1% FBS medium, then, DMSO, ROT, and ROA were added and incubated further for 48 h. Floating cells in cell culture medium were harvested and combined with adherent cells, cell lysates were prepared. The bar graphs depict the ratio of p-Akt Ser473/β-actin (A; $F_{(8,18)} = 8.003$, $p = 0.0001$, $R^2 = 0.7805$), t-Akt/β-actin (B; $F_{(8,18)} = 0.1034$, $p = 0.9986$, $R^2 = 0.04395$), p-ERK Thr202,Tyr204/GAPDH (C; $F_{(8,18)} = 30.20$, $p < 0.0001$, $R^2 = 0.9307$), t-ERK/GAPDH (D; $F_{(8,18)} = 0.4297$, $p = 0.8889$, $R^2 = 0.1604$), p-SAPK Thr183,Tyr185/GAPDH (E; $F_{(8,18)} = 10.71$, $p < 0.0001$, $R^2 = 0.8264$), and t-SAPK/GAPDH (F; $F_{(8,18)} = 0.3198$, $p = 0.9479$, $R^2 = 0.1245$). Data are expressed as mean ± SEM with n=3 and analyzed by one-way analysis of variance (ANOVA) followed by Tukey's *post hoc* test. *p<0.05, **p<0.01, and ***p<0.001 significantly different from control cells.

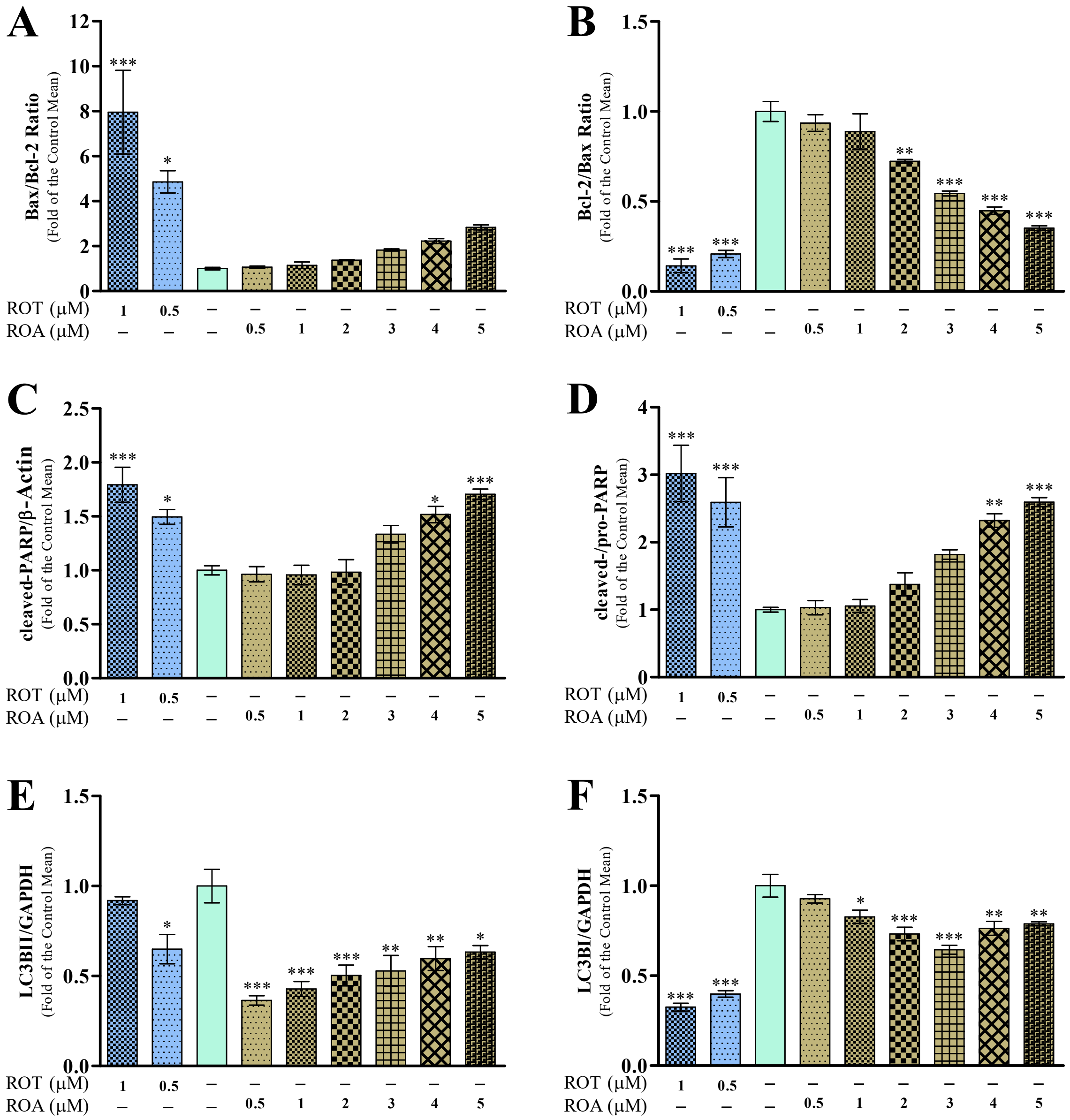

Supplementary Figure S7. SH-SY5Y cells were seeded at a density of 50,000 cells/ml in DMEM containing 1% FBS and incubated for overnight (~16 h). 95% of the medium was replaced with fresh DMEM + 1% FBS medium, then, DMSO, ROT, and ROA were added and incubated further for 48 h. Floating cells in cell culture medium were harvested and combined with adherent cells, cell lysates were prepared. The bar graphs depict the ratio of Bax/Bcl-2 (A; $F_{(8,18)} = 12.84$, $p < 0.0001$, $R^2 = 0.8509$), Bcl-2/Bax (B; $F_{(8,18)} = 52.65$, $p < 0.0001$, $R^2 = 0.9590$), cleaved-PARP/β-actin (C; $F_{(8,18)} = 13.79$, $p < 0.0001$, $R^2 = 0.8598$), cleaved/pro-PARP (D; $F_{(8,18)} = 14.76$, $p < 0.0001$, $R^2 = 0.8678$), LC3B II/GAPDH (E; $F_{(8,18)} = 11.64$, $p < 0.0001$, $R^2 = 0.8380$), and LC3B I/GAPDH (F; $F_{(8,18)} = 43.94$, $p < 0.0001$, $R^2 = 0.9513$) were assessed by Western blotting. Data are expressed as mean ± SEM with n=3 and analyzed by one-way analysis of variance (ANOVA) followed by Tukey's *post hoc* test. *p<0.05, **p<0.01, and ***p<0.001 significantly different from control cells.

Supplementary Table S1. Western blotting antibodies used in this study.

Antibody Name	Host, MW Details	Company	Cat. No.	Dilution
**Primary Antibodies:**				
Tyrosine Hydroxylase	Rabbit pAb, 62 kDa	Merck Millipore	AB152	1:1,000
DJ-1	Rabbit mAb, 22 kDa	Cell Signaling	#5933	1:2,000
p-α-synuclein (Ser129)	Rabbit mAb, 18 kDa	Abcam	ab51253	1:1,000
total α-synuclein	Rabbit mAb, 18 kDa	Abcam	ab212184	1:1,000
Neuronal Nuclei	Rabbit mAb, 46~55 kDa	Cell Signaling	#24307	1:1,000
Neurofilament-H	Mouse mAb, 180~200 kDa	Cell Signaling	#2836	1:1,000
β3-tubulin	Rabbit mAb, 55 kDa	Cell Signaling	#5568	1:1,000
p-Akt (Ser473)	Rabbit pAb, 60 kDa	Cell Signaling	#9271	1:1,000
t-Akt	Rabbit pAb, 60 kDa	Cell Signaling	#9272	1:1,000
p-ERK1/2 (Thr202/Tyr204)	Rabbit mAb, 44,42 kDa	Cell Signaling	#4370	1:1,000
t-ERK1/2	Rabbit mAb, 44,42 kDa	Cell Signaling	#4695	1:1,000
p-SAPK (Thr183/Tyr185)	Rabbit mAb, 46,54 kDa	Cell Signaling	#4668	1:1,000
t-SAPK	Rabbit mAb, 46,54 kDa	Cell Signaling	#9252	1:1,000
Bax	Rabbit pAb, 23 kDa	Santa Cruz	sc-493	1:1,000
Bcl-2	Rabbit mAb, 26 kDa	Cell Signaling	#4223	1:1,000
Mcl-1	Rabbit mAb, 40 kDa	Cell Signaling	#94296	1:1,000
Caspase-9	Mouse mAb, 47,37,35 kDa	Cell Signaling	#9508	1:1,000
Caspase-3	Rabbit mAb, 35,19,17 kDa	Cell Signaling	#14220	1:1,000
Caspase-7	Rabbit mAb, 35,20 kDa	Cell Signaling	#12827	1:1,000
PARP	Rabbit pAb, 116,89 kDa	Cell Signaling	#9542	1:1,000
LC3B	Rabbit mAb, 16,14 kDa	Cell Signaling	#3868	1:1,000
p62 (SQSTM1)	Rabbit mAb, 62 kDa	Cell Signaling	#39749	1:1,000
BiP (GRP78)	Rabbit pAb, 78 kDa	Cell Signaling	#3183	1:1,000
GAPDH	Rabbit mAb, 37 kDa	Cell Signaling	#2118	1:3,000
GAPDH (HRP conjugate)	Rabbit mAb, 37 kDa	Cell Signaling	#8884	1:3,000
β-actin (HRP conjugate)	Rabbit mAb, 45 kDa	Cell Signaling	#5125	1:3,000
**Secondary Antibodies:**				
Anti-rabbit IgG, HRP-linked antibody		Cell Signaling	#7074	1:1,000
Anti-mouse IgG, HRP-linked antibody		Cell Signaling	#7076	1:1,000

p-, phosphorylated; t-, total.
pAb, polyclonal antibody; mAb, monoclonal antibody; kDa, kiloDalton.

Fig. 3A

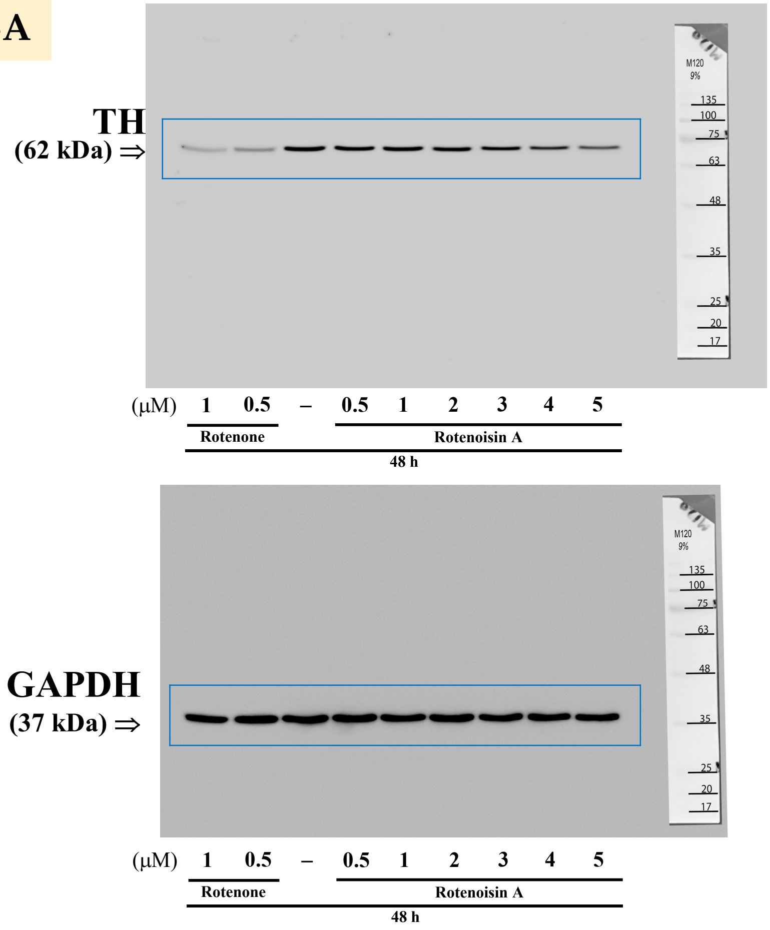

Fig. 3B

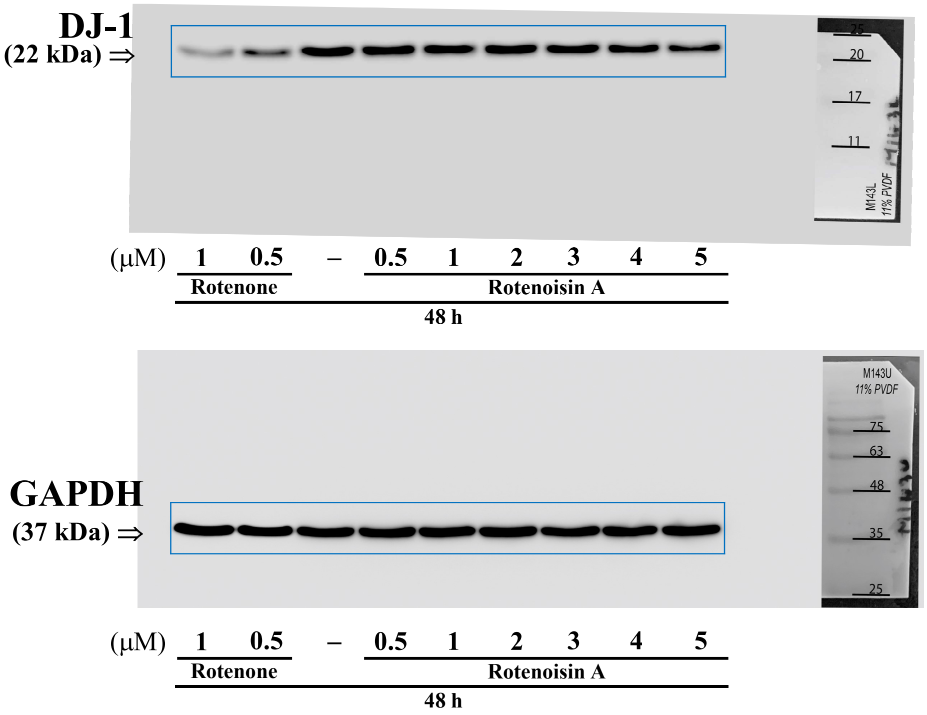

Supplementary Figure S8. Unedited chemiluminescence images from Western blots used in Figure 3 of this manuscript shown with their reference molecular weight markers. Blue colored brackets indicate the appropriate molecular weight bands of each target protein.

Fig. 4A

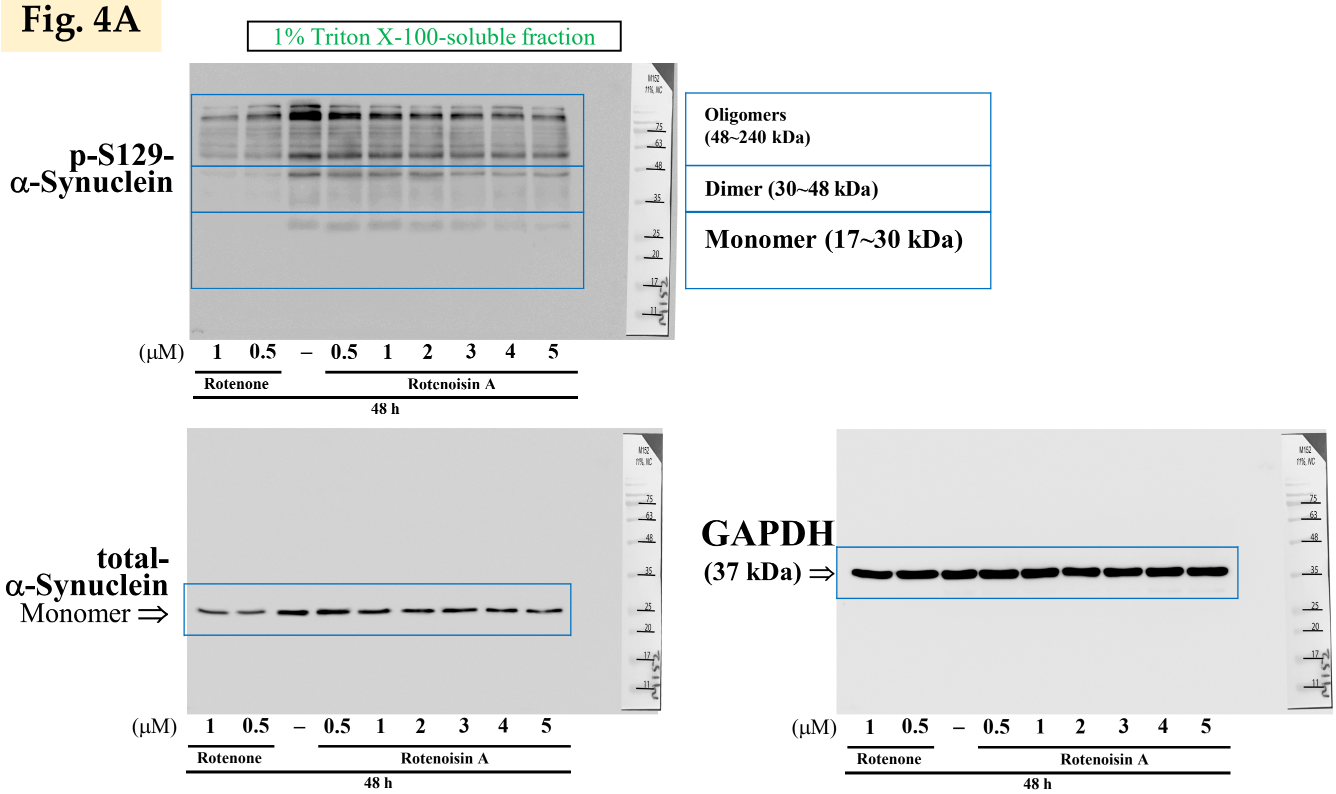

Fig. 4C

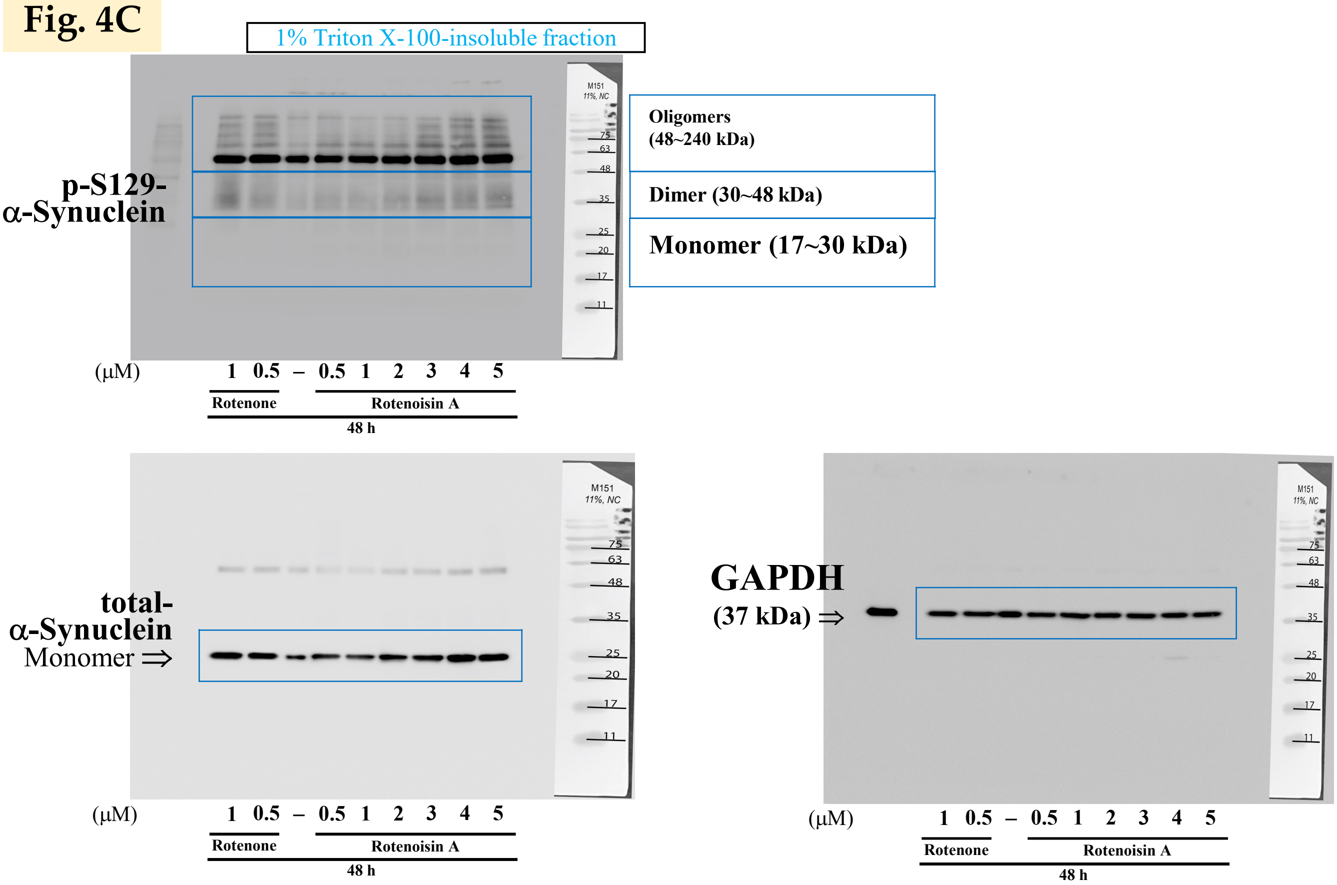

Supplementary Figure S9. Unedited chemiluminescence images from Western blots used in Figure 4 of this manuscript shown with their reference molecular weight markers. Blue colored brackets indicate the appropriate molecular weight bands of each target protein.

Fig. 5A

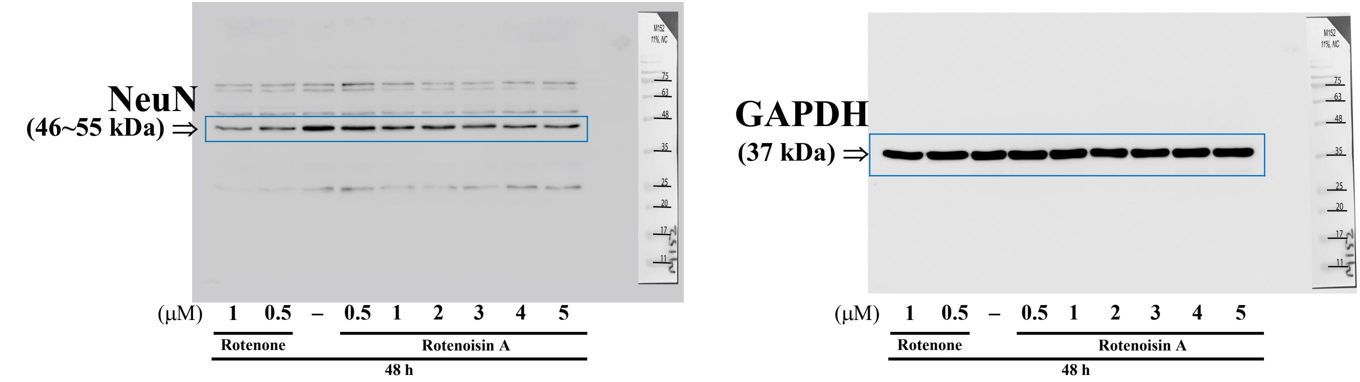

Fig. 5B

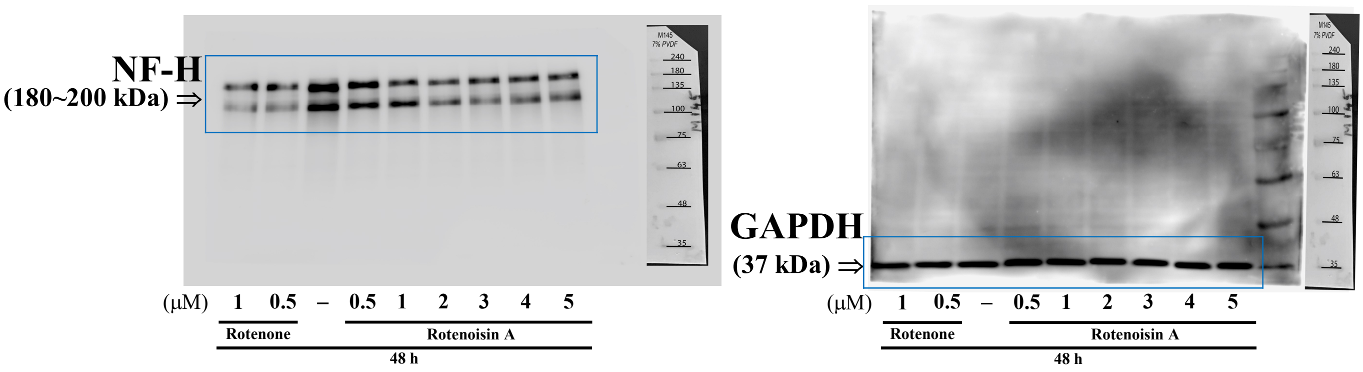

Fig. 5C

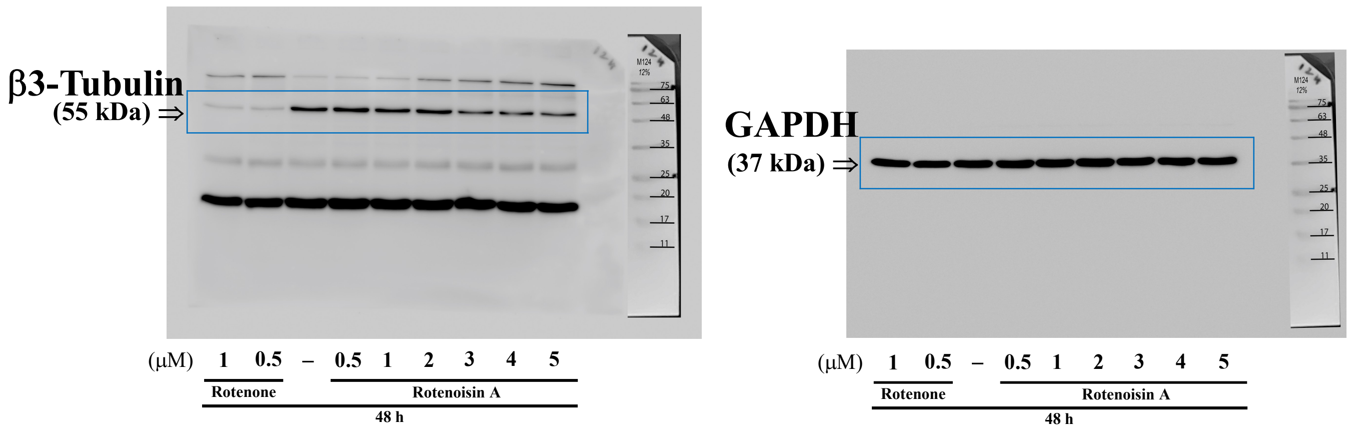

Supplementary Figure S10. Unedited chemiluminescence images from Western blots used in Figure 5 of this manuscript shown with their reference molecular weight markers. Blue colored brackets indicate the appropriate molecular weight bands of each target protein.

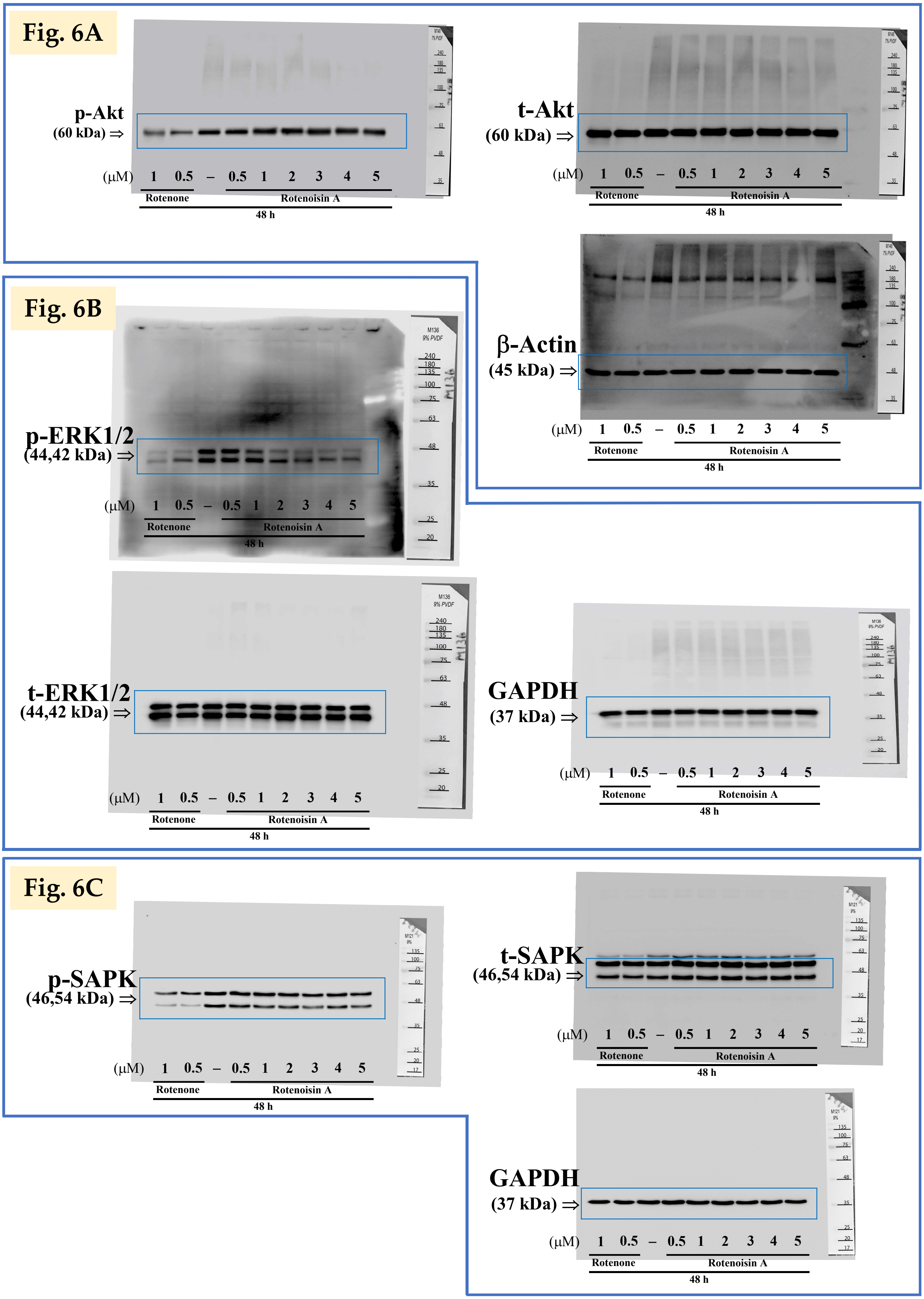

Supplementary Figure S11. Unedited chemiluminescence images from Western blots used in Figure 6 of this manuscript shown with their reference molecular weight markers. Blue colored brackets indicate the appropriate molecular weight bands of each target protein.

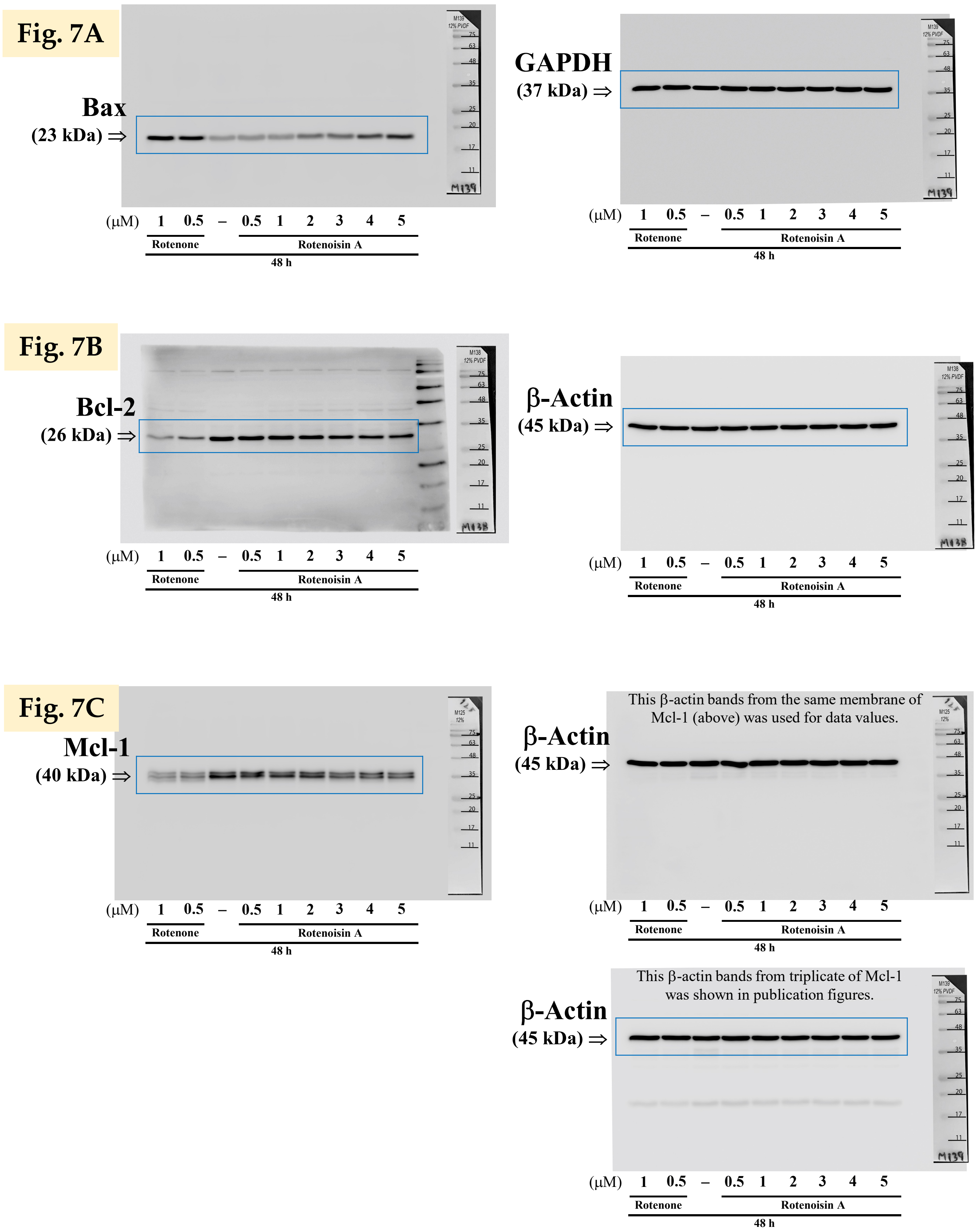

Supplementary Figure S12. Unedited chemiluminescence images from Western blots used in Figure 7 of this manuscript shown with their reference molecular weight markers. Blue colored brackets indicate the appropriate molecular weight bands of each target protein.

Fig. 8A

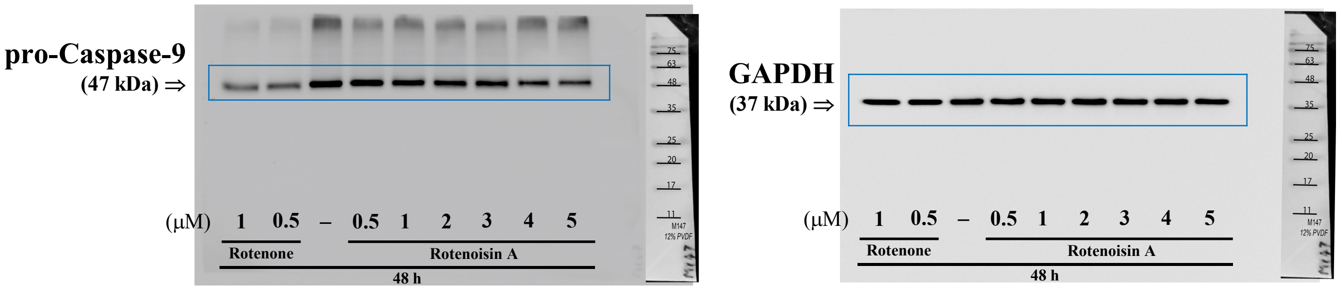

Fig. 8B

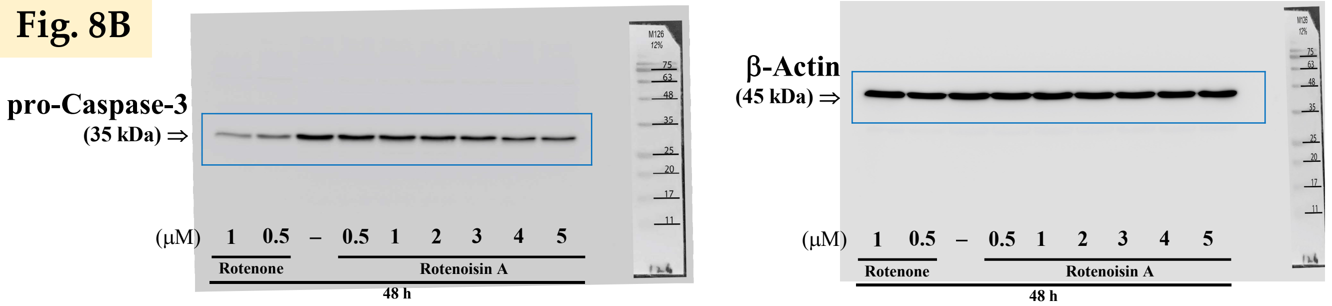

Fig. 8C

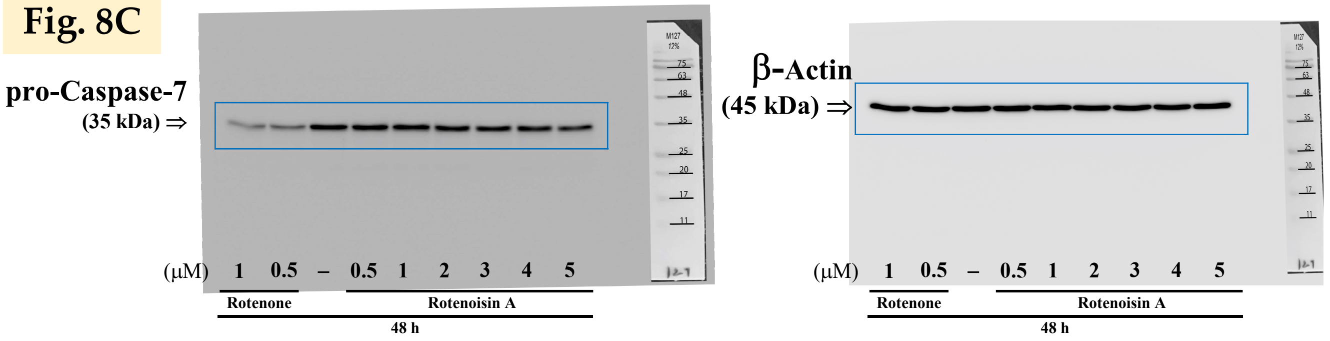

Fig. 8D

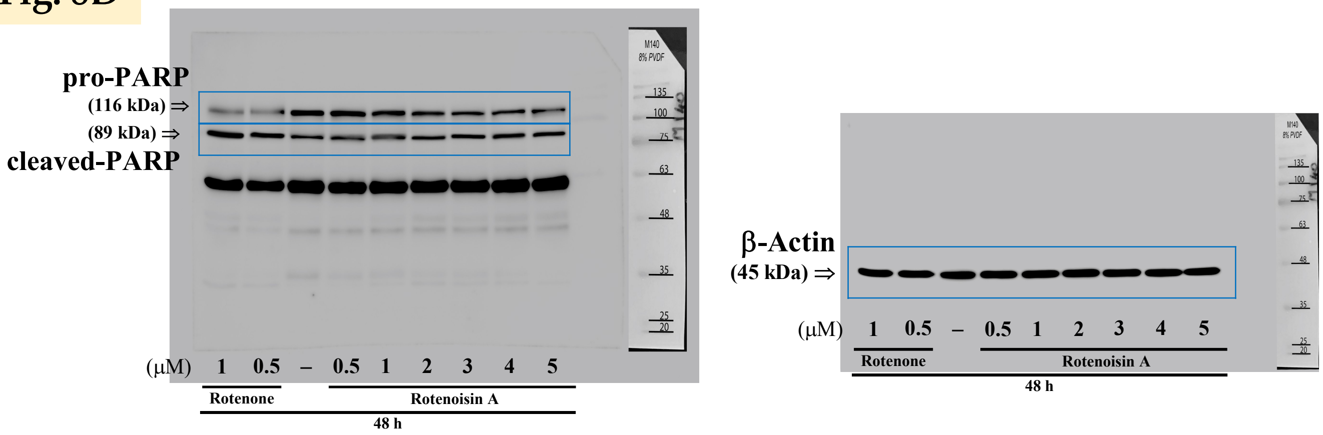

Supplementary Figure S13. Unedited chemiluminescence images from Western blots used in Figure 8 of this manuscript shown with their reference molecular weight markers. Blue colored brackets indicate the appropriate molecular weight bands of each target protein.

Fig. 9A

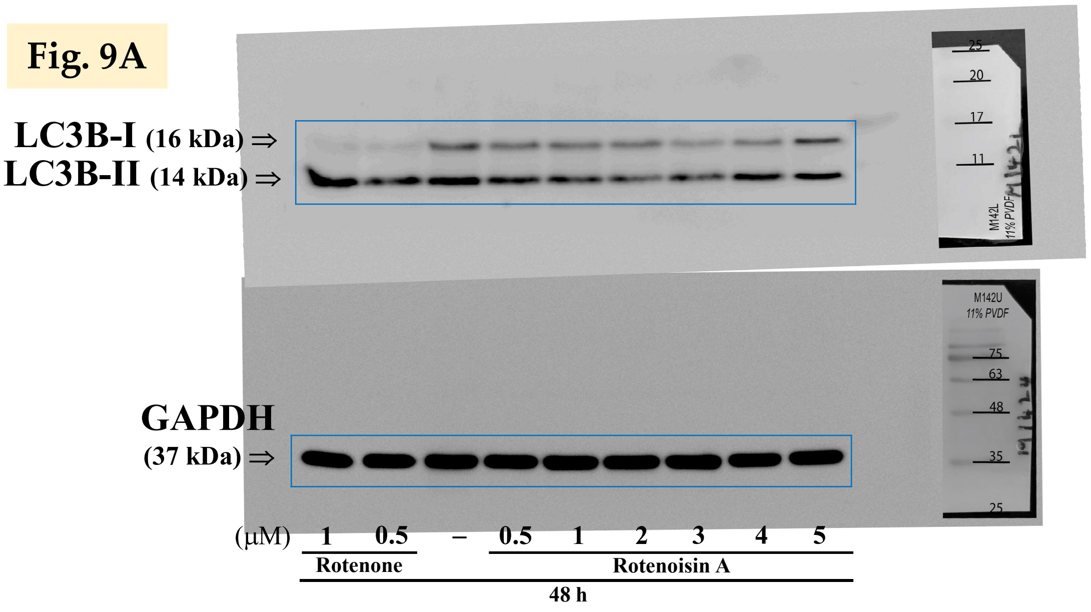

Fig. 9B

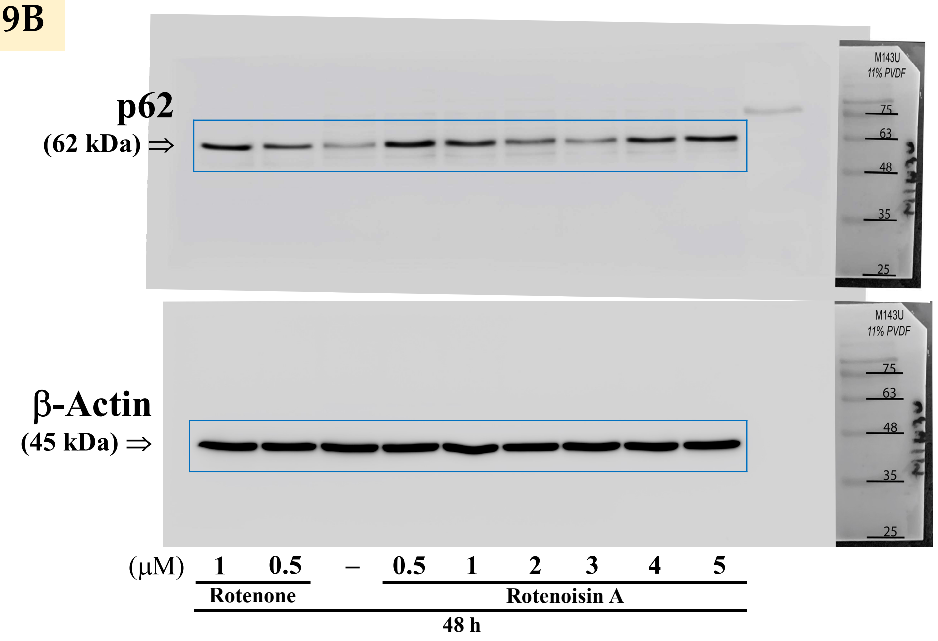

Fig. 9C

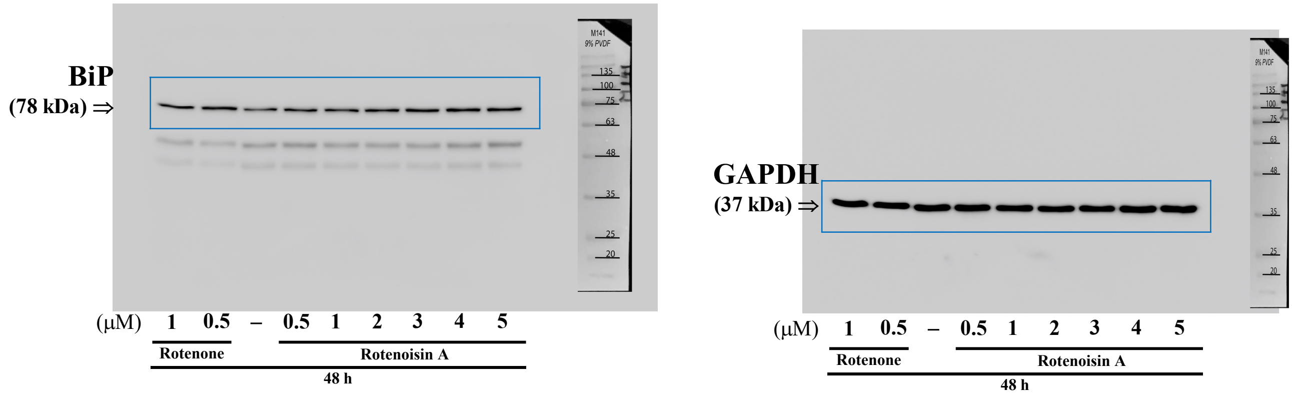

Supplementary Figure S14. Unedited chemiluminescence images from Western blots used in Figure 9 of this manuscript shown with their reference molecular weight markers. Blue colored brackets indicate the appropriate molecular weight bands of each target protein.
